# Single cell Hi-C identifies plastic chromosome conformations underlying the gastrulation enhancer landscape

**DOI:** 10.1038/s41467-023-39549-4

**Published:** 2023-06-29

**Authors:** Nimrod Rappoport, Elad Chomsky, Takashi Nagano, Charlie Seibert, Yaniv Lubling, Yael Baran, Aviezer Lifshitz, Wing Leung, Zohar Mukamel, Ron Shamir, Peter Fraser, Amos Tanay

**Affiliations:** 1 Department of Computer Science and Department of Biological Regulation, Weizmann Institute of Science, Rehovot, Israel; 2 The Blavatnik School of Computer Science, Tel Aviv University, Tel Aviv, Israel; 3 Laboratory for Nuclear Dynamics, Institute for Protein Research, Osaka University, Osaka, Japan; 4 Nuclear Dynamics Programme, The Babraham Institute, Cambridge, UK; 5 Department of Biological Science, Florida State University, Tallahassee, FL USA

**Keywords:** Embryology, Systems analysis, Epigenomics

## Abstract

Embryonic development involves massive proliferation and differentiation of cell lineages. This must be supported by chromosome replication and epigenetic reprogramming, but how proliferation and cell fate acquisition are balanced in this process is not well understood. Here we use single cell Hi-C to map chromosomal conformations in post-gastrulation mouse embryo cells and study their distributions and correlations with matching embryonic transcriptional atlases. We find that embryonic chromosomes show a remarkably strong cell cycle signature. Despite that, replication timing, chromosome compartment structure, topological associated domains (TADs) and promoter-enhancer contacts are shown to be variable between distinct epigenetic states. About 10% of the nuclei are identified as primitive erythrocytes, showing exceptionally compact and organized compartment structure. The remaining cells are broadly associated with ectoderm and mesoderm identities, showing only mild differentiation of TADs and compartment structures, but more specific localized contacts in hundreds of ectoderm and mesoderm promoter-enhancer pairs. The data suggest that while fully committed embryonic lineages can rapidly acquire specific chromosomal conformations, most embryonic cells are showing plastic signatures driven by complex and intermixed enhancer landscapes.

## Abstract (web summary)

Here the authors use single-cell Hi-C to investigate chromosome conformation in post-gastrulation mouse embryos. They find a distinct genome organization in primitive erythrocytes and conformations matching the mesodermal and ectodermal lineages.

## Introduction

The organization of mammalian chromosomes^1^ must accommodate physical nuclear packaging constraints alongside three major sources of dynamics – transcription^2^, replication^3^ and differentiation^4–6^. Recent advances in microscopy^7^, and different conformation capture technologies^8^ have provided improved understanding of the way chromosomes fold in general, leading to models for organization at multiple scales; from chromosomal territories and interchromosomal spaces^9^, through active and inactive (also known as A and B) intra-chromosomal compartments, and cohesin/CTCF mediated loop structures^10^. These models explain observations on the distribution of chromosomal contacts and domain insulation that give rise to topological associated domains (TADs)^11–14^. Moreover, parallel advances in mapping the dynamics of genome replication show a high degree of linkage between chromosomal compartments, TADs, and genome replication time control^15,16^, highlighting genome replication as a key driver of the linkage between chromosomal structures and cellular proliferation. Quantification of the mitosis and replication cycle in chromosomes using synchronized cells^17,18^ and single cell Hi-C^19,20^ was used to combine the effects of mitotic compaction and genome replication into one model describing the effect of cellular proliferation on chromosomal structure. Overall, current data indicate that chromosomes are continuously being remodeled in all phases of the cell cycle - during exit from the mitotic state (M-G1 phase), while replicating (S-phase), and when re-entering the mitotic state (G2-M phase).

A cycling dynamics of chromosome structure is therefore unavoidable for proliferating cell populations. This dynamics can be challenging if cells should combine proliferation with the acquisition of stable transcriptional and epigenetic identities. A classical model for a process that must balance remarkable proliferation with rapid differentiation is embryonic development. Recent advances in single cell RNA-seq have provided unbiased and detailed maps of the earliest stages of transcriptional sorting during embryo gastrulation^21^. These data confirmed and refined the classical observations on the emergence of an epiblast cell population and its rapid diversification into the three germ-layers by embryonic day 7.5. It also showed that diversification within the germ-layer is rapid and almost immediate, including early expansion of embryonic blood and several distinct mesodermal lineages, the differentiation of basic ectodermal neuronal progenitors, and the emergence of endodermal precursors from primitive precursors and convergent extra-embryonic endoderm lineages^22^. Since these dramatic transcriptional events are occurring while cells are dividing at maximal rates (at least every 8 h on average), the chromosomal structure underlying them must simultaneously support replication and cell-fate acquisition. But it is currently not understood if and when chromosome conformation/structure in embryonic lineages differentiates and stabilizes. It is unclear if cell-type specific chromosomal structures that were observed in-vitro^23,24^ or in mature tissues^25,26^ emerge before cells establish transcriptional identities, during (and in direct correlation with) transcriptional sorting, or only several cell cycles after cells commit to their fate transcriptionally.

Here we use single cell Hi-C to explore the chromosomal organization of post-gastrulation embryonic cells. We developed algorithms that combine analysis of replication time traces with contact distributions to enable de-novo clustering of single cells in the embryo while minimizing bias by cell cycle signatures. This leads to two main observations on the timing and structure of the initial cell type specific chromosomal structures in the embryo. First, we discover that a highly distinct chromosomal conformation is characterizing primitive erythrocytes, showing that in principle, conformation can be specified and stabilized rapidly in differentiated cell types in the embryo. In contrast to this effect, most of the embryo nuclei show much milder conformational heterogeneity that is associated primarily with broad clustering into mesoderm and ectoderm architectures. We show that the overall conformations of single cell Hi-C clusters representing the mesoderm and ectoderm layers are remarkably similar at the level of compartments and TADs. Nevertheless, we show that promoter-enhancer contacts that link ecto- or mesoderm specific promoter activity with specific enhancer markup are enriched for differential long-range contacts. Further analysis suggests that enhancers that are specific to diverse gastrulation lineages are interleaved within one group of TADs, while enhancers that are more accessible in the pluripotent epiblast state are demarcated from these genomic domains in a second group of TADs. Together the data suggest that while committed embryonic lineages may acquire specific chromosomal conformations rapidly, the majority of the embryonic lineages in gastrulation share a common and possibly more plastic chromosomal structure.

## Results

### Cell cycle signatures dominate embryonic chromosome conformations

**Fig. 1:**
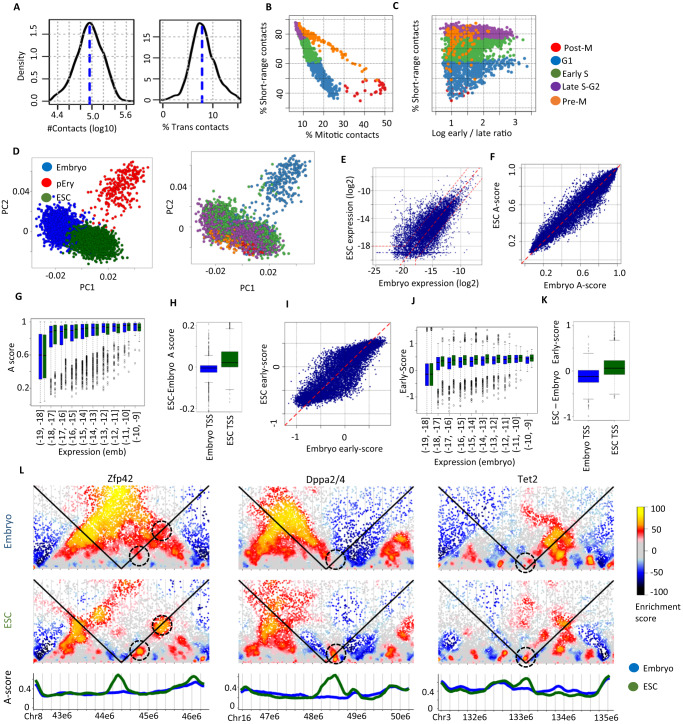
Single cell Hi-C in mouse embryo cells. **A** Distribution of the number of unique contacts per cell (left) and fraction of trans-chromosomal contacts per cell (right) in the Embryo scHi-C dataset. **B** For each single cell shown are the fraction of contacts in the 2Mb-12Mb (“mitotic”) distance band vs. the fraction of contacts between elements less than 2 Mb apart (“short-range”). Color coding is based on classification into cell cycle phases as in Nagano et al. 2017. **C** Comparing normalized ratio of scHi-C coverage on early and late replicating loci (X axis) to fraction of short-range contacts. **D** Visualizing clusters of single ES and embryo cells using PCA projection of A-scores from 11 genomic clusters. Cells are color coded according to cluster (left) or the initial annotation of cell cycle phase (right). **E** Plotting gene expression of 40 kb bins in ESCs compared to embryo cells (mean across E9 metacells, excluding pEry). Upper and lower dashed lines indicate the threshold for defining transcriptional changes between embryo and ESC. **F** Comparison of A-scores for 40 kb genomic bins. **G** 40 kb genomic bins were stratified according to embryonic expression level (units are log2 of the expression frequency). The distributions of A-scores in embryos (blue) and ESCs (green) are depicted using boxplots. The (−19, −18] box contains at least *n* = 48 K genomic bins, (−11, −10] and (−10, −9] at least *n* = 20, and the rest at least *n* = 200. Box limits are the first and third quartile, center line is the median, whiskers are 1.5 times the interquartile range, and points are outliers. **H** Distributions of differential A-score (ESC minus Embryo) in genomic bins with TSSs showing differential gene expression in embryos compared to ESCs (*n* = 1289 ESC induced bins: green, *n* = 806 Embryo induced bins: blue). Box limits are as in (**G**). **I**–**K** Similar to (**F**–**H**) but showing data on the early-scores of genomic bins instead of A-scores. **L** Examples of conformation reprogramming at pluripotency loci. For each locus we show Shaman enrichment plots in Embryos (top) and ESC (middle), and the respective A-score trends (bottom; blue – embryo, green – ESCs). Dashed circles represent focal points for differential conformation.

We applied single-cell Hi-C to assay chromosomal conformation in three E9.5 C57BL/6 J mouse embryos. We processed 3456 embryonic cells, out of which 87.15% passed quality control (QC) (Supplementary Fig. 1A–J). We sequenced at a depth that allowed recovery of a median of 91 K contacts per nucleus, with an overall low rate of trans-chromosomal contacts (median 7.85%), demonstrating high library quality (Fig. 1A). Across all cells, we captured 310 M contacts, with 8% trans-chromosomal contacts. We initially phased nuclei along the cell cycle using our previously reported strategy^19^, observing high degree of similarity between the parameters of the cell cycle model originally inferred for mouse embryonic stem cells (mESCs) and the embryonic cells. For example, we observed that 6.2% of the nuclei are enriched (20% or more) for contacts in genomic distances ranging between 2–12 Mb (Fig. 1B), defining a canonical mitotic cycle as previously observed for mESCs. Ordering embryo nuclei based on their distribution of contact distances as in Nagano et al.^19^ (Supplementary Fig. 1K) recapitulated the cell cycle dynamics involving transition between a G1 conformation landscape defined by long range (>12 Mb) contacts and the S-phase regime involving gradual increase in short range (<2 Mb) contacts. To allow robust comparison of the replication time trends between ESC and Embryos we identified genomic regions that are constitutively replicating early or late in S-phase according to both datasets (defined as “strict early” and “strict late”, Methods, Supplementary Fig. 2A, B). Analysis of the ratio between Hi-C coverage in these genomic regions in embryo cells showed partial consistency with the trend observed in ESC, where we observed an increase in the ratio through mid-S phase and a decrease toward G2 (Fig. 1C). Interestingly, in the embryo this trend was perturbed by a population of nuclei with high early/late coverage ratios and an atypically low fraction of short-range contacts in cells that were initially annotated as G1. These data reinforced our earlier observations on the dominance of cell cycle signatures in scHi-C, but also suggested the canonical signature may be shadowing additional conformational heterogeneity within the embryonic nuclei pool.

### Clustering scHi-C profiles using S-phase cluster seeding and RNA atlas projection

To enable de-novo clustering of scHi-C profiles with reduced cell-cycle bias, we developed a two-stage approach (denoted *S-phase cluster seeding*). We seeded scHi-C clusters using analysis of replication time trends in mid-S phase cells and expanded these seeds to clusters using A-compartment association scores (A-scores, Methods). We applied this approach to a combined data set of ESC and embryo cells (Supplementary Fig. 2C–H), deriving a model defined by three main clusters, one involving a distinct group of embryo nuclei with non-canonical cell cycle phasing (C3, Supplementary Fig. 2I), and the other two representing clustering of the remaining embryo (C2) and ES (C1) nuclei. As expected, M-phase nuclei were poorly separated into clusters, but otherwise G1-S cell cycle variation was captured as intra-cluster structure (Fig. 1D).

To annotate nuclei clusters and explore their underlying gene regulatory programs, we acquired and sequenced single cell RNA from two E9.0 embryos and from ESCs using MARS-seq and created a map of transcriptional states using Metacell^27^ (Supplementary Fig. 3A, B). We identified differentially expressed genes and genomic bins encompassing them for each expression metacell and projected scHi-C clusters on the transcriptional maps by calculating relative A-scores on these genomic bins. Remarkably, this strategy associated unambiguously C3 conformations with primitive erythrocyte (pEry) expression (Supplementary Fig. 3C), but showed that the remaining transcriptional landscape in the embryo could not be matched by strong conformation clusters within C2. This was further confirmed by re-analysis of a reference gastrulation scRNA-seq atlas (E6.5–8.25, Supplementary Fig. 3D, E). Overall, despite the rich transcriptional embryonic space, C2 nuclei were reflecting variation that was approximately similar in extent to the transcriptionally homogeneous ESC states represented in the C1 cluster and only primitive erythrocytes stood out as a distinct conformation cluster.

### Differential contacts in pluripotent and embryonic nuclei

Many genomic bins showed average transcriptional changes in non-pEry embryo cells compared to ESCs (806 and 1289 bins with over 4-fold decrease and increase respectively, Fig. 1E). Global comparison of *A-scores* in the ESC (C1) and embryo (C2) clusters (Fig. 1F) showed however conservation of the A/B compartment structure, with 85% of the genomic bins showing less than 0.1 change in A-score, and only 0.2% showing over 0.3 change. Analysis of A-score in loci stratified according to expression levels (Fig. 1G, excluding bins with differential expression) suggested a clear distinction between A-linked expressed and B-linked non-expressed loci. Further analysis also indicated that bins containing genes over expressed in the ESC or embryo will have a higher A-score in that sample (Fig. 1H, KS D = 0.4, *p* << 0.01). This association was observed based on relatively small changes in A-score and despite the lack of loci showing major A–B compartment switches. We next compared embryo and ESC estimated replication time per genomic bin (defined as the early-score, Methods). This suggested a similar trend of expression linkage (Fig. 1I–K, KS = 0.36, *p* << 0.01). Together these data show that despite the mild magnitude of compartment and replication-time remodeling in embryos compared to ESCs, it still reflects transcriptional regulation in these cells.

We next searched for localized differential chromatin contacts in ESCs and embryo cells by pooling contacts from single-cell clusters and performing Shaman^28,29^ normalization and enrichment analysis. Using a threshold of differential enrichment score of 50, we identified 3267 pairs of loci losing contacts and 1914 pairs of loci gaining contacts in embryos compared to ESCs, suggesting many cases of local conformation remodeling. We observed that genomic bins with higher A-score in ESCs are involved in significantly more differential contacts than loci with constitutively high A-score or loci gaining A-score in the embryo (Supplementary Fig. 4A, two-sided Kolmogorov–Smirnov test). A screen for differential contacts at ESC-regulated TSSs highlighted cases of conformation changes with potential regulatory impact (Supplementary Data 1). For example, we observed specific contacts and insulation structure isolating the pluripotency genes *Rex1/Zfp42*, *Tet2* and *Dppa2/4* from surrounding B-compartment associated regions in ESC nuclei (Fig. 1L, see Supplementary Fig. 4B for conformation changes in loci conserving their A-association). These data suggested that specific contacts, with possible linkage to gene regulation and in particular to the repression of the pluripotency program, are observed in embryonic nuclei. This is occurring even when global structural features such as compartment, replication and insulation (Supplementary Fig. 4C) are changing only mildly.

### Primitive Erythrocyte chromosomes show compact and highly organized folding

**Fig. 2:**
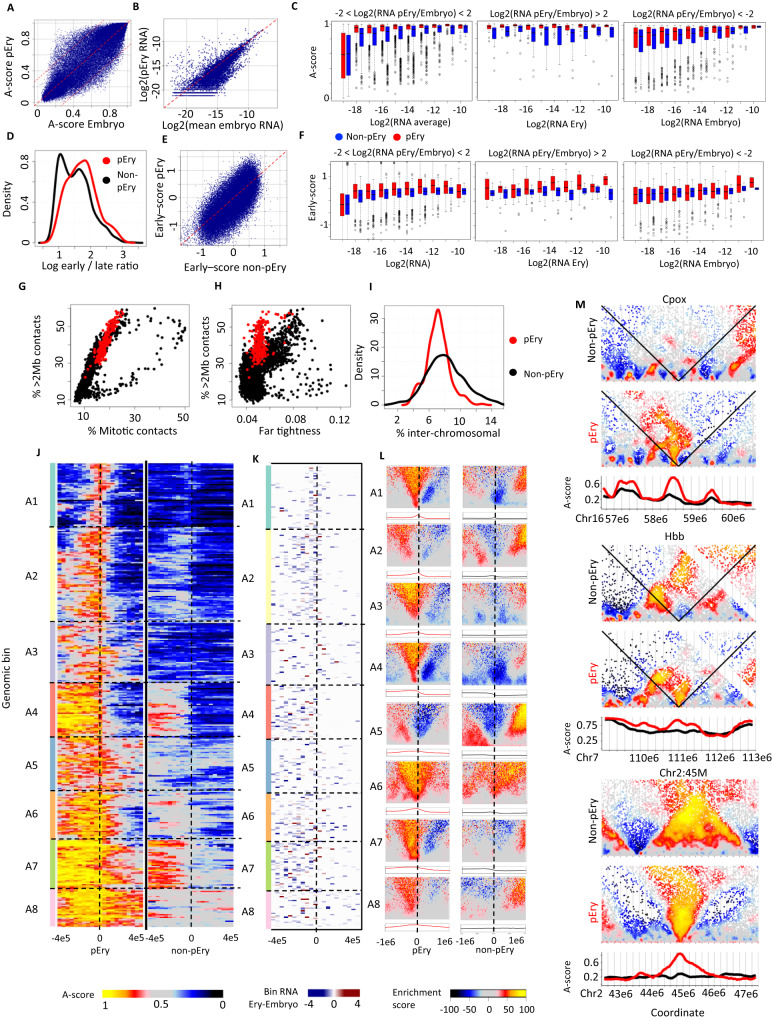
Distinct, compact conformation for primitive erythrocytes. **A** Comparison of 40 kb bins A-score in pEry cells vs. non-pEry embryo cells. Upper and lower dashed lines show differences of at least 0.3 in A-score. **B** Comparison of log2 mean expression (fraction of molecules per gene) for 40 kb genomic bins. **C** Distribution of genomic bins’ A-score as a function of expression levels. A-score and transcription were calculated for 40 kb genomic bins. Plots show A-scores stratified by expression, for loci classified with conserved expression (left), Ery induced expression (middle) and Ery-repressed expression (right). In the left panel, the (−19, −18] box contains at least *n* = 48 K genomic bins, (−11, −10] and (−10, −9] at least *n* = 20, and the rest at least *n* = 200. In the middle panel, all boxes contain at least *n* = 15 genomic bins, except for the (−11, −10] and (−10, −9] which contain at least *n* = 5. In the right panel, all boxes contain at least *n* = 90 genomic bins, except for (−12, −11], (−11, 10] and (−10, −9] which contain at least *n* = 35, 10, and 2 respectively. Box limits are the first and third quartile, center line is the median, whiskers are 1.5 times the interquartile range, and points are outliers. **D** Distribution of single cell early/late coverage ratio for pEry (red) and non-pEry (black) cells. **E** Comparing early-scores for 40 kb genomic bins in pEry and non-pEry embryo cells. **F** Similar to (**C**), but showing distributions of 40 kb genomic bins early-score instead of A-score. **G** Showing the distribution of contacts with distance >2 Mb vs mitotic contacts (2–12 Mb) in pEry (red) and non-pEry cells (black). Note the general high degree of long range contacts in p-Erys. **H** Showing the fraction of contacts in the most frequent distance bin (defined as “Far tightness” in Nagano et al 2017) compared to the rate of long-range contacts. **I** Distributions of inter-chromosomal contact rates for pEry and non-pEry cell. **J** Shown are color coded A-scores computed for the pEry (left) and non-pEry (right) clusters around loci with pEry specific high A-score (400 kb upstream and downstream). Loci are grouped into 8 clusters using K-means clustering. **K** For each of the loci clustered in J we color coded bins with any level of transcription according to the relative expression in pEry and non-pEry cells (blue – higher in non-pEry, red - higher in pEry). **L** Loci within each cluster in J were pooled, and their average Shaman score is color coded for pEry and non-pEry cells. The pooled A-score profile is shown at the bottom for every loci cluster in pEry and non-pEry. **M** Examples of loci showing distinct pEry conformation. For every locus, depicted are contact enrichment in non-pEry cells (top), pEry cells (middle) and profile of A-score in the two clusters (bottom). For *Cpox* and *Hbb* we mark contacts with the TSS locus by black diagonal lines.

In contrast to the weak separation of scHi-C clusters C1 and C2, the pEry cluster C3 was defined by a well separated group of 264 single cells (reclassified using total A-scores per cell, Supplementary Fig. 4D). This separation was supported by a large number of genomic bins with modified A-score in pEry compared to other embryo cells (Fig. 2A, 4.8% with A-score delta > 0.3). We estimated mean expression in pEry and non-pEry E9 embryo metacells (Fig. 2B) and noted that in pEry, genomic bins bearing expressed genes at any level show remarkable alignment to the A-compartment (Fig. 2C). Estimation of single cell early/late coverage ratios (Fig. 2D), showed that cells classified as pEry are enriched in S-phase, but are also represented in other phases. Genome replication landscapes (quantified by early-scores) were more conserved than A-scores (Fig. 2E), but genomic bins containing expressed genes showed earlier replication, in concordance with their increased A-score (Fig. 2F). Beyond its unique compartment structure, the pEry single cell cluster was also characterized by uniformly high fractions (30–60%) of contacts over >2 Mb (Fig. 2G). The data also showed high variance for pEry long range contact distances, with no distance bin representing over 6% of the contacts (Fig. 2H). This property distinguishes the long-range contacts in pEry maps from those observed in embryonic or ESC G1 cells during exit from mitosis. Despite the higher rate of long range intra-chromosomal contacts, pEry nuclei show low rates of trans-chromosomal contacts (Fig. 2I). Together these observations indicate pEry chromosomes form compact and highly organized territory structures, with A/B compartments that are strongly demarcated and reflective of transcriptional activity patterns.

### pEry funnel-like A-compartment structures are anchored at TSSs and cryptic loci

To understand further the sharp increase in pEry A-compartment association specificity, we identified 357 loci with the highest increase in pEry specific A-scores. Clustering of A-scores profiles over 400 kb around such pEry A-specific loci showed that about 50% of the sites (Fig. 2J, clusters A1-A3) involved sharp A-linked pEry hotspots that reside in the B compartment in non-pEry embryo cells. The remaining sites typically represented increase in A-score for a larger domain bounded by the identified pEry A-linked peak (clusters A4-A8). Projection of differential TSS expression on the clustered genomic interval confirmed that the majority (92%) of pEry A-peaks were associated with an expressed TSS (Fig. 2K). Interestingly it also suggested many hotspots of A-association could not be explained by any known localized transcriptional driver. We then computed the mean pEry and non-pEry contact enrichment patterns for the loci clusters (Fig. 2L). In pErys, this revealed an unexpected trend involving a funnel-like structure representing aligned contacts around the focal A-compartment contact hotspot. Contact enrichment maps around the same loci in non-pEry nuclei showed these sites are located within embryo insulators and between two loop structures (Fig. 2L - right). We visualized individual loci showing major funnel-like conformational remodeling around key genes (Fig. 2M) and multi-peak loci (Supplementary Fig. 4E), but also in hotspots that represented uncharacterized regulatory effects (Fig. 2M, bottom). This suggested that strong A-compartment alignment in pErys is not driven solely by transcription, and must therefore also involve some other trans-acting factors (e.g., we observed enrichment for erythrocyte TF binding, Supplementary Fig. 4F, G). For control, we clustered profiles of 272 loci with top non-pEry A-score increase, indicating lack of similar funnel-like effects in the embryo conformation cluster (Supplementary Fig. 4H). To validate that the highly specific conformations in C3 nuclei are indeed representing primitive erythrocytes in a non-biased fashion, we sorted directly 118 primitive erythrocytes cells from E10.5 embryos and generated new single-cell Hi-C profiles from them (Supplementary Fig. 5A–D). The data confirmed that sorted pEry cells represent the same sharp A/B compartment structure as the one characterized in non-sorted cells and reconfirmed the presence of remarkable funnel-like structures in these cells (Supplementary Fig. 5E, F). Of note, Guo et al. recently reported a similar funnel structure in thymocytes and B cells, which they termed “chromatin jets”, suggesting its prevalence in hematopoietic cells^30^.

### Refined embryo clustering by model-based analysis of replication dynamics

**Fig. 3:**
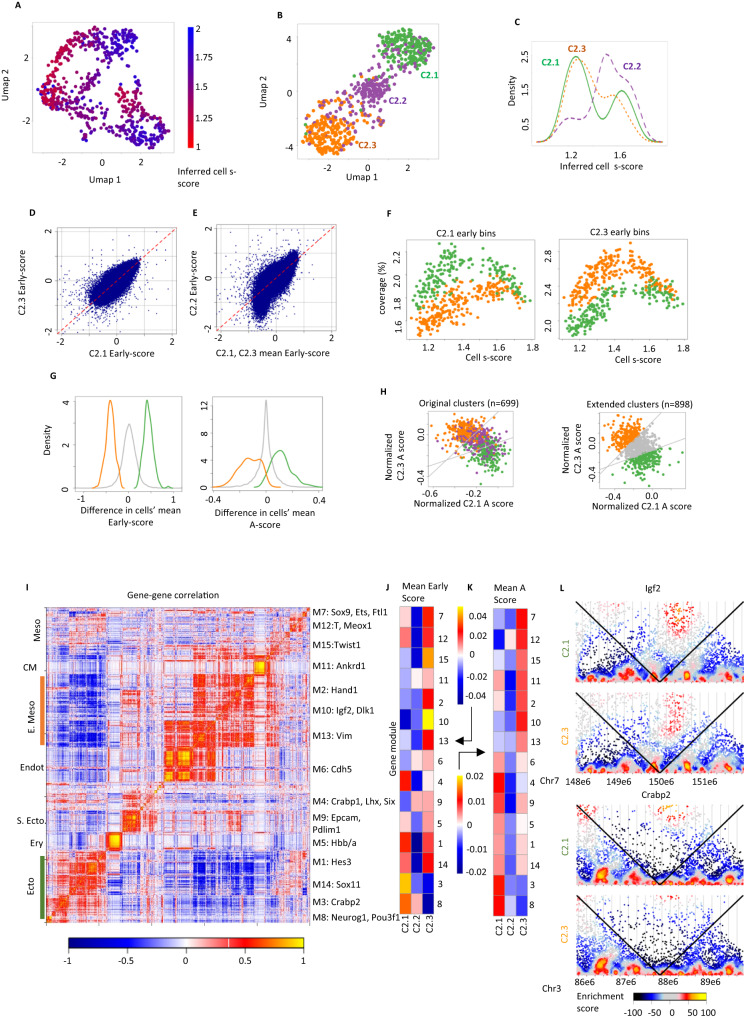
Ectoderm and mesoderm/endoderm scHi-C clusters in the embryo. **A** S-phase cells from the non-pEry cluster were identified and projected on 2D using UMAP analysis of their coverage in 1103 loci. Cells are color-coded by their s-score as inferred by our probabilistic model. **B** UMAP projection of the same cells as in (**A**), using features normalized given inferred s-score for each cell. **C** Distribution of inferred s-scores for the three non-pEry embryo clusters. **D** Average normalized coverage (early-score) for genomic bins in clusters C2.1 and C2.3. **E** Similar to (**D**), but comparing average C2.1 and C2.3 behavior to C2.2 behavior. **F** Genomic bins that were inferred to be early replicating (Methods) in C2.1 (left) or C2.3 (right) were pooled, and for each cell we plotted total coverage as a function of the inferred s-score. Cells are colored by their cluster (C2.1 – green, C2.3 – orange). **G** Distribution of the difference between C2.1 cells and C2.3 cells in early-score (left) and A-score (right) for genomic bins classified as specific to C2.1 (green) or C2.3 (orange). Grey – all bins. **H** Average normalized A-score for the group of genomic bins specific to C2.1 (X) and C2.3 (Y) are depicted for color-coded cells in the three clusters C2.1- 3 (left). A Similar plot is shown for 898 cells that were not included in the set of 699 mid S-phase cells used for clustering (right). Gray lines mark the thresholds used for classification of the expanded C2.1 and C2.3 clusters. **I** Correlation heatmaps for 2353 gene expression profiles over the E9.0 metacell model. Gene module numbers and representative genes are shown on the right. S. ecto Surface ectoderm, CM cardiomyocyte, Endot Endothelium, E Meso extraembryonic mesoderm. **J** The color-coded matrix represents the difference in average early-score per single cell cluster (columns) for the TSS loci in each gene module from I (rows). **K** Similar to (**J**), but showing difference in average A-score in each cluster. **L** Depicting the contact structure (color-coded Shaman map) in C2.1 (top) and C2.3 (bottom) cells around the Crabp2 and Igf2 TSSs.

Since embryo transcriptional states are highly heterogeneous at E9, we made several attempts to enhance resolution within cluster C2, searching for conformation variation that can be linked with differentiating cell types on the background of massive proliferation signatures. Direct clustering of single cell coverage profiles in S-phase cells and UMAP visualization of these cells (Fig. 3A) suggested cell-to-cell variation may be present in the data, but showed that it is superimposed over strong cell-cycle gradients, even when restricting analysis to replicating cells alone. We therefore developed a sensitive algorithm that considers both the replication cycle and the potential cell-type structure explicitly and quantitatively (Supplementary Fig. 6A, B). The algorithm infers a probabilistic mixture model in which each cell is associated with a cluster and a latent replication timing variable defined as the *s-score*. Each cluster specifies the replication timing of each genomic bin, such that once a cell’s s-score is inferred, the algorithm can compare its observed bins read coverage to the values predicted by a linear replication process that is timed in a bin-specific way (Methods). The algorithm tries to fit the observed data by clustering cells de-novo while simultaneously inferring their s-scores and the cluster-specific replication timing parameters. We used cross-validation to tune model parameters and verify the algorithm robustness (Supplementary Fig. 6C–I). This resulted in good matching of observed and modeled replication regimes (Supplementary Fig. 6J) for a model including 3 clusters denoted C2.1, C2.2 and C2.3. Importantly, the model’s inferred s-scores facilitate normalization of the coverage statistics for each cell. UMAP projection of such normalized profiles show a clear, cell-cycle independent cluster structure (Fig. 3B). Analysis of the observed cluster structure suggested C2.1 and C2.3 are distributed homogeneously along the replication cycle (Fig. 3C). Cluster C2.2 showed skewed distribution enriched for late-S profiles and additional analysis indicated cells within the cluster are of lower coverage and potentially lower quality (Supplementary Fig. 7A). We note that we could not derive robust results using alternative methods for clustering scHi-C data, which are based on differential compartment structure and are lacking explicit cell cycle modelling^31,32^ (Supplementary Fig. 7B, C).

### Ectoderm and mesoderm/endoderm scHi-C clusters

We estimated replication time per genomic bin (early-score) in the C2.1-3 clusters to facilitate their further annotation. These estimations were consistent for C2.1 and C2.3 (Fig. 3D), but showed C2.2 cells are skewed to high and low coverage values (as expected by their bias to mid to late-S phase, Fig. 3E). We identified groups of genomic bins with C2.1 or C2.3-specific early replication, showing that pooling coverage on these groups provided replication-time dependent separation of the clusters (Fig. 3F). Moreover, mean A-score over the same genomic bin groups showed matching separation of single cells (i.e. early replicating loci in C2.1 were also more A-associated in C2.1 and conversely for C2.3, Fig. 3G). This allowed expansion of our clustering to additional S-phase cells (Fig. 3H). A similar approach was not applicable to G1 cells (Supplementary Fig. 7D). Overall this strategy yielded a total of 431 C2.1 cells and 504 C2.3 cells for further analysis. We searched for cluster-specific replication time in groups of loci representing correlated gene modules inferred from scRNA-seq data (Fig. 3I–K, Supplementary Fig. 7E–G). This unambiguously associated cells in cluster C2.1 with ectoderm gene expression programs and cells in C2.3 with mesoderm or endoderm programs. Cells in C2.2 were not associated with any gene expression program. The annotation of clusters C2.1 and C2.3 was supported by comparing their compartments to data from Neural Progenitor Cells (NPCs) and Hematopoietic Stem Cells (HSCs) from E14.5 embryos (Supplementary Fig. 7H–J)^24,33^.

### Lineage-specific scHi-C conformation differences are weak

To test the potential for detecting additional cell-type structure given the limited breadth and depth of our scHI-C sample, we performed simulations with downsampled data. These experiments show that the data and algorithms are sufficiently sensitive to allow detection of clusters similar to C2.1 and C2.3 even when these involved as little as 50–75 cells (~5–10% of the modeled cells, Supplementary Fig. 8A, B). This suggests that other possible chromosomal differences between cell types are weaker, or are present in scarcer cell populations. Further analysis suggested that even for the mesoderm and ectoderm clusters, contact landscapes could be remarkably similar, even around loci that support dramatic transcriptional regulation (e.g., *Igf2* or *Crabp2*, Fig. 3L). Quantitatively, only 1% of the genome (divided into 40 kb bins) show A-score different of 0.2 or more between the clusters, compared to 3.6% in a comparison of the E14.5 NPC and HSC maps (Supplementary Fig. 8C).

Consistent with their overall similarity in conformation landscapes, further dissection of the mesoderm or ectoderm into cell types using our mixture model approach (Supplementary Fig. 8D, E) was deriving only cell-cycle dependent refinements of the C2.1 and C2.3 clusters. To improve on this, we used inferred replication time parameters to normalize coverage profiles per cell in each cluster (Supplementary Fig. 9A, B). Hierarchical clustering of the resulted data did identify an intra-mesoderm lineage structure, including a small cluster strongly matching the endothelial transcriptional state (Supplementary Fig. 9C, D). It can therefore be hypothesized that replication time and compartment structure of refined embryonic lineages may be detected using sensitive algorithms and deeper single cell sampling. But the data strongly suggest that the magnitude of conformational changes between such refined lineages will remain small, in particular compared to the highly distinct pEry state we described above.

### Three-way identification of regulated long-range interactions

**Fig. 4:**
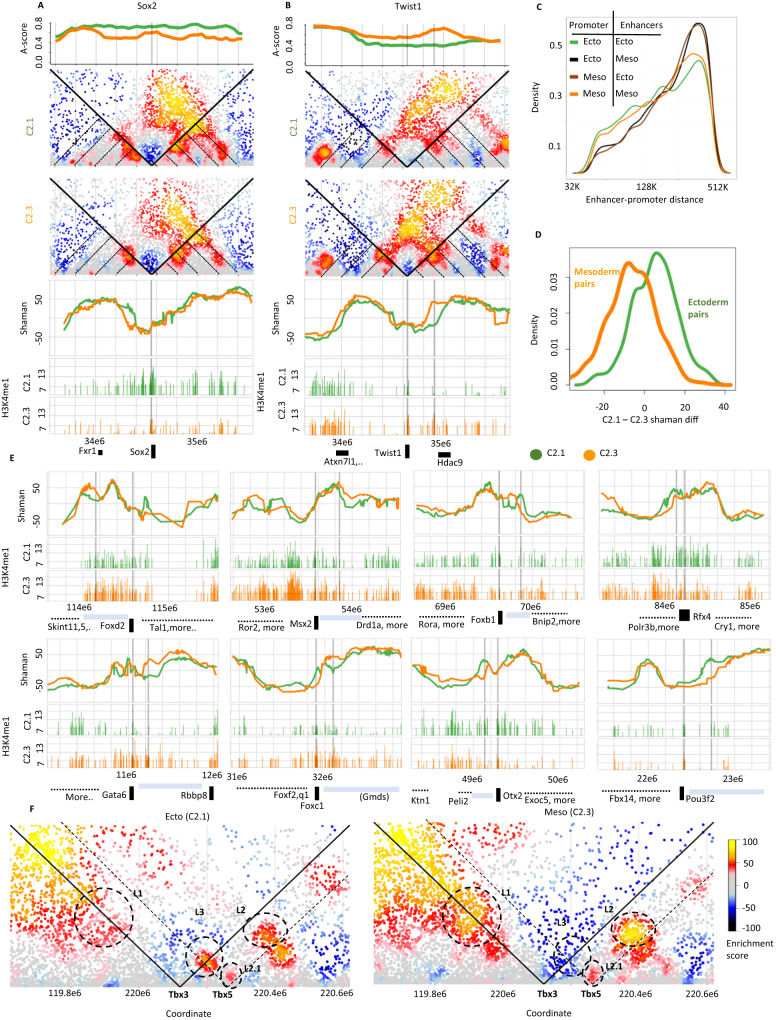
Three-way support for specific regulatory contacts. **A**, **B** Comparing A-score (top), contact maps, virtual-4C using Shaman scores, and H3K4me1 ChIP-seq (bottom) around the *Sox2* and *Twist1* loci. The genes, and for *Twist1* also a nearby enhancer, are marked by vertical grey lines. **C** Shown are distributions of genomic distances between a TSS and the nearest putative enhancer classified according to the ectoderm/mesoderm lineage specificity of the two loci as determined by gene expression (for the promoter) and ENCODE ChIP-seq (for the putative enhancer). **D** The distribution of differential C2.1 and C2.3 Shaman score (X axis) on TSS-enhancers pairs with coordinated mesoderm or ectoderm specific activity. Shaman differences is computed only for contacts with positive scores in both C2.1 and C2.3. **E** Examples of virtual 4 C plots (top) and H3K4me1 ChIP-seq (bottom, C2.1 followed by C2.3) around 4 ectoderm and 4 mesoderm genes. Gray vertical lines mark the TSS and putative enhancer. Gene-free regions around regulated genes are highlighted by horizontal gray bars. **F** Contact structure around the *Tbx3*-*Tbx5* locus in the C2.1 and C2.3 clusters. Contacts discussed in the text are marked by dashed circles.

Pooling contacts in ectoderm and mesoderm scHi-C clusters provided us with a strategy for identifying germ-layer specific chromatin interactions. We first identified 256 and 236 loci with higher ectoderm or mesoderm/endoderm A-score respectively. Annotation of these sites (Supplementary Data 2) revealed several important regulatory genes, for example the mesoderm TF *Twist1*, and the epiblast/ectoderm TF *Sox2* (Fig. 4A, B). Comparative analysis of contact maps in these loci showed again a very high degree of consistency between the global conformation of the two clusters. Nevertheless, we could identify refined alteration in contact distributions of the promoter of *Twist1* with a putative regulatory element (shown by virtual 4C, Fig. 4B). To generalize this observation, we used published histone modification maps from ectoderm (hind-, mid- and forebrain) and mesoderm (heart, limb) tissues, and identified cell type specific putative enhancers (Methods, Supplementary Fig. 10A, B). We also screened for identified genes with germ-layer specific expression, and combined them with the epigenomic maps by mapping each enhancer to its closest promoter. Proximal pairs of enhancers and promoters with matching ecto- or mesoendo- specific activity could then be defined (Fig. 4C). To complete a three-way integrative screen on putatively interacting pairs, we next computed the contact enrichment in the C2.1 and C2.3 contact maps for each of the matching promoter-enhancer pairs. We observed significant contact enrichment in the ectoderm scHi-C cluster for ectoderm specific promoter-enhancer pairs, and mesoderm contact enrichment in the mesoderm pairs (Fig. 4D). We also implemented a direct statistical test for contact frequency around putative ectoderm and mesoderm hotspots (Supplementary Fig. 10C, D), which supported a similar observation. We note that the two methods differ in their normalization strategy and power, and their identified hits are only partly overlapping. When using Shaman comparison, we detected 173 and 338 promoter-enhancer pairing with 3-way support for ecto- and mesoderm regulatory activity respectively (Supplementary Data 3), including many examples linked with key regulators of cell type specific transcriptional programs (See examples in Fig. 4E). These putative interactions should be interpreted carefully. First, while we believe comparisons using Shaman scores are more sensitive, these cannot be fully controlled statistically. Second, we note that only 1.5% of the highest intensity (Shaman score difference > 40) differential ectoderm-mesoderm contacts were annotated within one of our enhancer-promoter pairs, illustrating that the complex conformational landscape in these clusters involves many uncharacterized contacts despite showing only weak compartment and TAD differences.

### Polycomb markup and ectoderm specific long-range contacts in the *Tbx*3-5 locus

Our 3-way analysis of regulated promoter-enhancer pairs suggested contact enrichment is positively linked with lineage-specific gene activation in most cases. It is however possible that contact enrichment will be associated with gene repression, as postulated previously for Polycomb domains^34–36^. We therefore screened for ectoderm/mesoderm differential H3K27me3 loci (using hind-, mid- and forebrain/heart and limb) with proximal anti-correlated promoter expression pattern (Supplementary Fig. 10E–G). This screen yielded several candidate locus pairs showing high H3K27me3 occupancy in correlation with proximal gene repression and low contact intensity (Supplementary Data 4), where most of these cases were of lower specificity than the positive interactions observed for activated genes. A reciprocal effect was detected in the *Tbx3*-*Tbx5* locus, where Polycomb marks and gene repression were associated with increased rather than decreased contact intensity. This locus codes for two transcription factors with sophisticated transcriptional control, where *Tbx3* is expressed in the epiblast and most mesodermal tissues, and *Tbx5* is specific to pharyngeal mesoderm and cardiomyocytes (Supplementary Fig. 10H). In the mesoderm cluster, consistently with previous reports^37^, we observed two TAD structures (contacts over L1 and L2, Fig. 4F, Supplementary Fig. 10I) physically separating the two TFs. In the ectoderm, however, the near-complete repression of both genes is correlated with the emergence of a new *Tbx3*-*Tbx5* contact (L3), and severe attenuation of the L1 contact. The internal structure at *Tbx5* (L2.1) is unperturbed. While we have not observed other repressive chromatin structures of similar intensity, this example suggests that de-novo establishment of chromatin interactions may be facilitated in the context of either the Polycomb or some other uncharacterized repressive machinery.

### Gastrulation cell-type specific accessibility hotspots are intertwined within TADs

**Fig. 5:**
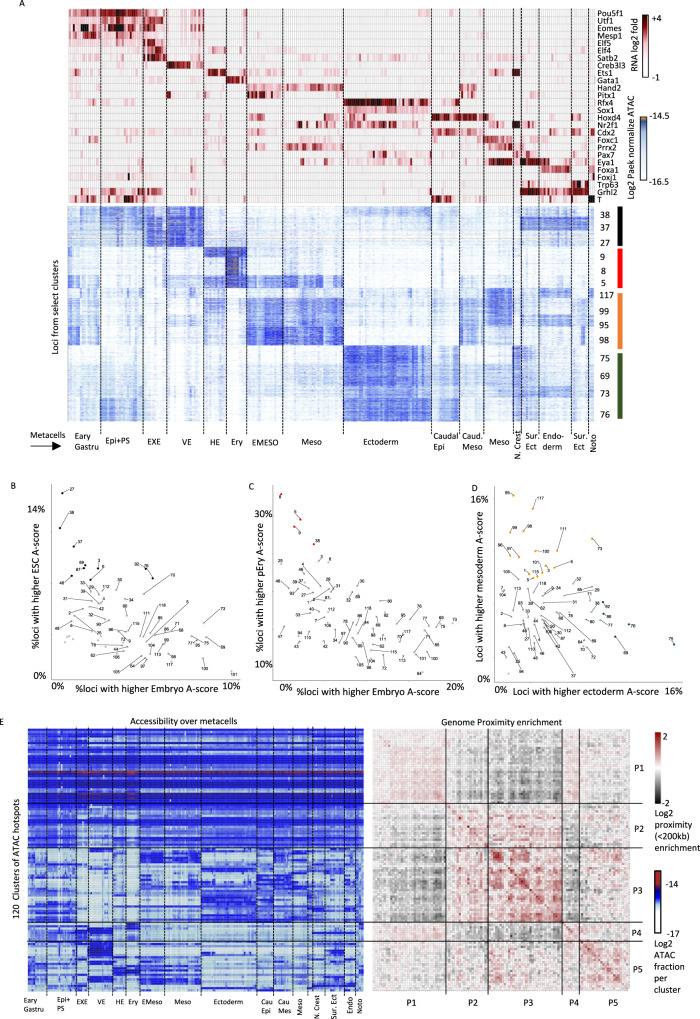
Gastrulation accessibility hotspots are chromosomally intertwined. **A** Bottom panel shows the accessibility of peaks (rows) over metacells (columns) (log2 the number of normalized ATAC-seq reads). Shown are loci from select clusters highlighted in the text. Top panel depicts gene expression of correlated TFs over the same metacells, provided in order to link accessibility clusters with specific cell types. **B** For each cluster of ATAC peaks we computed the fraction of loci with A-compartment score difference larger than 0.1 when comparing ESC and Embryo pooled Hi-C. Clusters with over 0.08 of the loci showing A-score enrichment in ESCs are colored black. **C** Similar to (**B**), but comparing embryo and pEry pooled Hi-C maps. **D** Similar to (**B**), but comparing the embryonic clusters C2.1 (ectoderm) and C2.3 (mesoderm). **E** Left panel is showing mean normalized accessibility for ATAC peak clusters (row) and metacells (column). Right panel is showing for each pair of peak clusters the enrichment of intra-TAD proximity (number of pairs of peaks in the same TAD and within 200 kb of each other).

We reasoned that the linkage between extensive transcriptional diversification in gastrulation and the rather rudimentary observed chromosomal conformation diversity must involve the chromosomal and genomic distribution of active regulatory elements and promoters. Using single-cell ATAC/RNA-seq multiomics data^38^, we derived clusters of cell type specific chromosome accessibility peaks with specific distributions over the key gastrulating cell types (Fig. 5A, Supplementary Fig. 10J). We then tested the A-score distribution of the loci in each cluster of peaks. Comparing ESC and embryo A-scores (Fig. 5B) we discovered stronger A-linkage in ESC for cluster 27, 37 and 38, which are enriched for accessibility in extraembryonic tissues and early gastrulation state (e.g. Epiblast). Comparing embryo and pEry A-scores (Fig. 5C) showed strong pEry A-linkage in clusters 8, 9 and 5, which represent erythrocyte or combined hematoendothelial peak specificity. Importantly, the extent of A-association differential for pEry clusters was significantly higher than that observed between ESCs and embryo cells. Comparison of mesoderm and ectoderm A-scores (Fig. 5D) showed several clusters with compatible A-score and accessibility preferences including clusters 69, 75 and 76 for the ectoderm, and clusters 95, 98, 99 and 117 for the mesoderm. This analysis also highlighted more complex combinatorics such as the one observed for cluster 73 (accessible in both ectoderm and endoderm).

The compartment association analysis of the ATAC peak clusters confirmed that we can observe strikingly cell-type specific accessibility hotspots in loci with very mild compartment association differences. Since chromosomal organization is observed at scales of at least 10 s of kilobases and TADs are typically organizing hundreds of kilobases into looped units, we reasoned that this effect could be explained if accessible hotspots with differential cell type activity were intertwined within large chromosomal units rather than demarcated into cell type specific domains. To test this idea, we computed log enrichment ratios for genomic proximity between clustered ATAC peaks. These values are positive if ATAC peaks from one cluster are more likely than expected by chance to be localized within 200 kb of peaks from another cluster in the same TAD. Negative values represent under-representation of pairs from the same cluster at <200 kb distance and within the same TAD. As shown in Fig. 5E, this analysis showed that peak clusters with activity in embryonic cell types but not extra-embryonic types (P2), or peak clusters with strong embryonic cell type specific accessibility (P3) are overall demarcated from constitutively accessible sites (P1) or loci that are active specifically in the extra embryonic or early embryonic states (P4). While there are additional proximity relationships within the embryonic peak clusters, the primary organizational principle seems to package the thousands of regulatory elements driving gastrulation in relative proximity, while isolating them from pluripotency or constitutive regulatory elements.

## Discussion

In order to characterize how chromosome conformations are reorganized immediately following gastrulation, we generated single cell Hi-C maps from more than 3000 mouse E9.5 embryo cells. We modeled the derived maps along two major axes: first, we aimed to account for the conformation changes occurring during the replication and mitotic cycle; second, we searched for clusters of conformations that can be associated with the rich transcriptional landscape in the embryo at this stage. Separating these two simultaneous dynamics in the embryo (or other tissues) remains a major analytical challenge. Identification of cell types in cells approaching mitosis or exiting it is not realistic at this stage. But algorithms we introduce here can use robust changes in genome replication time to cluster mid S-phase cells and then derive contact matrix-based (in particular differential A-compartment association) signatures from S-phase clusters. Based on these signatures, cells from nearly all parts of the cell cycle can be classified into balanced models of cell types. Once a cluster structure is inferred, we can pool contacts from single cells into conformation maps and explore cluster-specific differential compartments, long-range contacts and putative promoter-enhancer interactions at high resolution.

Our analysis of Hi-C maps in mouse post-gastrulation highlights several aspects of the relationship between chromosome conformation and embryonic differentiation. First, while the genome organization of ES cells compared to the embryo reflects changes in regulation of key pluripotency genes, the organization within the embryo is largely homogeneous. This suggests that differences in chromosomal conformation between ES cells and E9.5 cells are greater than those between different cell types immediately after gastrulation and at the onset of organogenesis. The exceptions to this homogeneity within the embryo are the distinctively folded primitive erythrocytes. Erythrocytes are unexpected positive controls for the ability to precisely detect a cell type specific conformation when it exists. The unique, compact and highly organized structure of pEry chromosomes cannot be explained by gene expression alone. In contrast to other differentiating embryonic tissues that continuously respond to signals from neighboring cells and tissue contexts, erythrocytes are fully committed to their functional fate, which may explain their highly distinct (and potentially less plastic) conformation. It is unclear if the erythrocyte chromosome condensation and enucleation program^39^ is related to the conformation we observe at E9.5, since definitive erythrocytes only appear several days after. Similar effect could be expected in other terminally differentiated cell types, such as cardiomyocytes or endothelium. But our analysis could not detect a cardiomyocyte conformation cluster, and the small cluster that we linked with endothelial programs could not be associated with a highly distinctive conformation, but was clustered as part of the mesoderm state. It is possible that the reason for this is that these cell states are differentiating much later than pEry cells.

Within the embryo-proper we detected two clusters that match broad ectoderm and mesoderm/endoderm genome regulatory programs. The considerable transcriptional diversity within the mesoderm (and to a lesser extent the ectoderm and endoderm at E9) at this stage was correlated very weakly with conformation sub-clusters within these two clusters. Our current scHi-C data is limited in its depth and number of cells, in particular compared to scRNA-seq or scATAC modern datasets and our analysis suggests that sampling more embryonic cells may lead to characterization of additional statistically significant conformation clusters. But subsampling and in-depth analysis show that such potential additional conformation clusters are unlikely to represent high intensity differential conformation features (as those we detected for pEry cells). The differences between the clusters in terms of replication time regime and compartment structure were small and we had to use sensitive algorithms to deconvolve them from the more apparent cell-cycle signature. Interestingly, on the background of such a homogeneous conformation landscape we detected hundreds of lineage specific promoter-enhancer contacts that showed matching expression and epigenetic markup in the respective tissues. This argues for an important role for localized embryonic contacts within an initially homogeneous TAD and compartment structure in the embryo. However, the epigenetic stability of such local contacts and the existence of factors regulating them (in addition to the known TFs binding the relevant enhancers) are still unclear. Furthermore, only a small fraction of differential contacts could be explained by enhancer-promoter interactions. It also remains to be seen how specific localized contacts and their higher order structures^29,40,41^ contribute to later emergence of broader contact structures, as previously observed in the brain and other tissues. Conversely, since we showed in the case of erythrocytes that chromosomes can in principle be reprogrammed quickly, it will be interesting to understand how conformation in the embryo remains relatively homogeneous despite the activity of specific gene regulatory program, which epigenetic factors may facilitate the maintenance of such a flexible conformation, and whether this is linked with the retained developmental plasticity of most embryonic cells at this stage.

## Methods

### Experimental methods

#### Cell extraction, fixation and permeabilization

Pregnant C57BL/6 mice were sacrificed at day 9.5 post-coitum and three embryos were dissected under a microscope, in accordance with the Babraham Institute Animal Welfare and Ethical Review Body. The yolk sack was mechanically removed from each embryo, leaving the embryo proper only, and the embryos’ morphology was validated to match that of a wildtype E9.5 embryo. To create single-cells suspension, each embryo was moved to a 1.5 ml tube containing 200 µl of trypsin-EDTA (0.05% trypsin, 0.02% EDTA) and incubated at 37 °C for 5 min. 800 µl of cold MEF medium was then added to each tube to inactivate the trypsin.

To fix the cells, the cell suspensions of all three embryos were combined and MEF medium at room temperature was added to a final volume of 21 ml. 3 ml of 16% formaldehyde were added (2% formaldehyde final concentration) and the mixture was incubated for 10 min at room temperature, followed by quenching with 127 mM glycine for 5 min on ice and washing with cold PBS + 0.001% BSA. Cells were then permeabilized in 10 mM Tris-Cl pH 8, 10 mM NaCl, 0.2% IGEPAL CA-630 and cOmplete EDTA-free protease inhibitor cocktail (Roche) for 30 min on ice with intermittent agitation, and spun to collect a nuclei pellet.

#### Single-cell Hi-C library preparation

scHi-C libraries were prepared in a fashion similar to the one previously described^19^. Briefly, the nuclei were washed with 1.24x NEBuffer 3 (New England Biolabs) and suspended in 400 µl of that buffer. 6 µl of 20% SDS and then 40 µl of 20% Triton X-100 were added to the suspension, with an incubation of 60 min at 37 °C with constant agitation following the addition of each of these detergents. Next 50 µl of 25 U/µl MboI (New England Biolabs) was added and the suspension incubated at 37 °C overnight with constant agitation.

To label the digested DNA ends, dCTP, dGTP, dTTP and biotin-14-dATP (Thermo fisher) were added to the suspension (final concentration of 28.36 µM per nucleoside triphosphate) along with DNA polymerase I, large (Klenow) fragment (New England Biolabs, final concentration 0.095 U/µl) and the sample incubated at 37 °C for 60 minutes with occasional mixing. The sample was then spun and the supernatant partially removed, leaving a volume of 50 µl, followed by the addition of 100 µl 10x T4 DNA ligase reaction buffer (New England Biolabs), 10 µl 100x BSA (New England Biolabs), 10 µl of 1 U/µl T4 DNA ligase (Thermo Fisher) and water to a final volume of 1 ml, and incubated at 16 °C overnight. Finally, the nuclei were filtered through a 30 µm cell strainer and single nuclei were sorted into individual empty wells in 384 well plates using an BD Influx cell sorter. The plates were sealed and stored at −80 °C until further processing.

To prepare single-cell Hi-C libraries from single nuclei in plate wells, 2.5 µl of PBS was added to each well and the plate was sealed and incubated at 65 °C overnight. DNA was then tagmented using the Nextera XT kit (Illumina) by adding 5 µl of TD and 2.5 µl of ATM per well and incubating at 55 °C for 5 minutes, followed by cooling to 10 °C and adding of 2.5 µl of NT per well. Hi-C ligation junctions were then captured by Dynabeads M-280 streptavidin beads (Thermo Fisher; 10 µl of original suspension per well). Beads were prepared by washing with 1x BW buffer (5 mM Tris-Cl pH 7.5, 0.5 mM EDTA, 1 M NaCl), resuspended in 4x BW buffer (20 mM Tris-Cl pH 7.5, 2 mM EDTA, 4 M NaCl; 4 µl per sample), and then mixed with the 12.5 µl per-well sample and incubated at room temperature overnight with gentle agitation. The beads were then washed four times with 40 µl of 1x BW buffer, washed twice with 40 µl of 10 mM Tris-Cl pH 7.5 and resuspended in 12.5 µl of 10 mM Tris-Cl pH 7.5. Single-cell Hi-C libraries were amplified from the beads by adding 7.5 µl of Nextera PCR Master Mix, 2.5 µl of Index 1 primer and 2.5 µl of Index 2 primer (a different combination of index 1 and index 2 per well) followed by 12 PCR cycles. The beads were then magnetically removed and the supernatant from all 384 wells combined. The combined supernatant was purified using AMPure XP beads (Beckman Coulter; 0.6 times volume of the supernatant) according to the manufacturer’s instructions and resuspended in 100 µl of 10 mM Tris-Cl pH 7.5. Finally, the sample was purified again using AMPure XP beads (1.0 times volume of supernatant) and resuspended in 11 µl of 10 mM Tris-Cl pH 7.5.

#### Embryo dissection and collection of primitive erythrocytes

Pregnant females were anesthetized with isoflurane using the open-drop system, followed by decapitation in accordance with a protocol approved by the Florida State University Animal Care and Use Committee (ACUC). Uterine horns were removed, rinsed in room temperature PBS and embryos were isolated and transferred to a droplet of DMEM-high glucose, 10% FBS, 2 mM L-glutamine, 1X MEM-Eagle non-essential amino acids and 12 ug/mL heparin (Sigma #H31493). Placenta and extraembryonic tissues were removed, embryos were decapitated and circulating peripheral blood was allowed to flow into the droplet of the room temperature media from the severed vitelline and umbilical veins. Media was collected, pooled, and brought to a volume of 21.875 mL with room temperature media. Cells were fixed by adding a final concentration of 2% paraformaldehyde for ten minutes. Fixation was quenched by bringing the solution to a final concentration of 0.127 M glycine, then incubating on ice for 5 min. Cells were pelleted, washed with PBS and pelleted. Cells were flash-frozen and kept at −80 C. Cells were thawed and stained for CD71 and TER119. Cells were first blocked with 1 mL of PBS-FT (5% FBS, 0.1% Tween-20) for 1 h, then stained with 1:200 anti-CD71-PE (Invitrogen, 12-0711-82) and 1:200 anti-TER119-APC (Invitrogen, 17-5921-82) for 2 h at room temperature. Cells were washed and resuspended in 500 uL PBS-F (2% FBS) and Hoechst (15 ug/mL) and subjected to FACS by Aria (BD Biosciences). Primitive erythrocytes (CD71+, TER119+) were collected and pooled into a 50 mL falcon for scHi-C processing following the established protocol (Nagano et al., 2017).

#### MARS-seq

MARS-seq on E9.0 embryos was performed as previously described^42^ sorting 15 plates from 2 129S4/SvJae embryos and sequencing a total of 5760 cells, out of which we retained for analysis 4781 cells with at least 1000 unique molecular identifiers (UMIs) each (median coverage 4574 UMIs). The experiment was performed in accordance with the institutional animal care and use guidelines of the Weizmann Institute of Science.

### Sequencing and basic computational analysis

#### scHi-C sequence processing, quality control and cell cycle phasing

We processed the scHi-C data as described previously^19^. Briefly, paired-end reads were demultiplexed to single cells using cell specific barcodes. Reads were broken to segments using matches to MboI recognition site (GATC), and segments were mapped to the genome using Bowtie2. Duplicate contacts were discarded.

We next performed quality control (QC) on each single cell. Cells were filtered based on their coverage (total number of reads), fraction of non-digested contacts, maximal chromosomal coverage aberration, and the contact distance bin with highest number of contacts.

To partition cells into different phases of the cell cycle, and order the cells within the phases, we calculated for each cell the fraction of “near” reads (with distance <2 Mb), the fraction of mitotic reads (with distance 2–12 Mb), mean contact distance for distances at least 4.5 Mb, and the fraction of contacts from a predefined set of early replicating regions. These statistics were used to phase cells into post-mitotic, G1, early to mid-S, mid-S to G2 and pre-mitotic phases, and to order cells within each phase. We note that this approach to phasing was only used as a preliminary stage for the algorithms described below.

#### Metacell analysis

We applied the Metacell algorithm^27^ to organize E9 single cell profiles in 77 metacells (excluding 69 outlier cells), that we summarized into quantitative expression profiles and visualized as previously described^27^. We also downloaded published single cell profiles from the mouse gastrulation atlas^21^ and generated 1306 atlas metacells on 110,291 QC-positive cells. Atlas metacells were annotated by majority voting on the published annotations of their cells, defining for each metacell *m*, the function atlas.type (*m*). Each atlas metacell i defined a gene expression distribution ${e}_{{gi}}^{{{{{{\rm{atlas}}}}}}}$ over the set of the 2237 feature genes g used while constructing the metacell graph. For annotation of the E9 map, we identified for each E9 single cell profile the atlas metacell with maximal correlations ${{{{{{\rm{ann}}}}}}}={{{{{\rm{atlas.type}}}}}}\left(\right.{{{{{{\rm{argmax}}}}}}}_{i}[{{{{{\rm{cor}}}}}}(\log ({u}_{g}+1),\,\log ({\epsilon }+{e}_{gi}^{atlas})])$, where ${u}_{g}$ is the UMI vector for the E9 cell and $\epsilon={10}^{-5}$ is a regularization factor. We then annotated each E9 metacell with the atlas annotation atlas.type that was linked with most of its cells.

#### Definitions and derivation of the strict early and strict late genomic subsets

We partitioned the genome into bins of size 200 kb (or 40 kb, depending on application) and counted scHi-C coverage per bin and cell in a matrix. We performed down-sampling of the scHi-C data such that each cell has 75k contacts and defined:

$${{{{{\rm{DSN}}}}}}={{{{{\rm{ds}}}}}}{{{{{{\rm{n}}}}}}}_{j}^{i}$$

as the number of contacts that map to genomic bin *j* in cell *i* after downsampling.

We next identified strict-early and strict-late genomics bins. This was done by clustering the genomic bins *j* using the vectors ${{{{{{\rm{dsn}}}}}}}_{j}^{i}$ into 4 groups using hierarchical clustering. The two clusters showing the highest and lowest coverage were shown to represent the previously observed^19^ A and B compartment structures respectively. These clusters behaved consistently (e.g. show enrichment (for A) and anti-enrichment (for B) in S-phase cells) between the pool of embryo and ESC cells. We will denote that derived genomic bins subsets ${{{{{{\rm{early}}}}}}}^{{{{{{\rm{strict}}}}}}}$ and ${{{{{{\rm{late}}}}}}}^{{{{{{\rm{strict}}}}}}}$.

We defined the early/late ratio of a cell as:

$${{{{{\rm{e}}}}}}{{{{{{\rm{l}}}}}}}^{i}={{\log }}2\left(\frac{\mathop{\sum}\nolimits_{j\in {{{{{{\rm{early}}}}}}}^{{{{{{\rm{strict}}}}}}}}{{{{{{\rm{dsn}}}}}}}_{j}^{i}}{\mathop{\sum}\nolimits_{j\in {{{{{{\rm{late}}}}}}}^{{{{{{\rm{strict}}}}}}}}{{{{{{\rm{dsn}}}}}}}_{j}^{i}}\right)$$

and classified mid S-phase cells as:

$${K}^{S}=\{i\,s.t.\,e{l}^{i}\, > \,1.8\}.$$

${K}^{{non}-S}$ was defined as all other cells.

#### A-score and early-score for genomic bins

For each genomic bin *j* and each cell *i*, we count the number of long range intra-chromosomal contacts (>1 Mb) observed between fragment ends in the bins and fragment ends in the ${{{{{{\rm{early}}}}}}}^{{{{{{\rm{strict}}}}}}}$ and ${{{{{{\rm{late}}}}}}}^{{{{{{\rm{strict}}}}}}}$ genome compartments, defining count vectors $c{A}_{j}^{i}$ and $c{B}_{j}^{i}$.

The A-score of a genomic bin is determined given a set *C* of scHi-C profiles (possibly all) as:

$${{{{{{\rm{scoreA}}}}}}}_{j}^{C}=\mathop{\sum }\limits_{\left\{i\in C\right\}}c{A}_{j}^{i}/\left[\mathop{\sum }\limits_{\left\{i\in C\right\}}c{A}_{j}^{i}+\mathop{\sum }\limits_{\left\{i\in C\right\}}c{B}_{j}^{i}\right].$$

The early-replication score (shortened early-score) of a bin is computed given a group *C* of cells (typically all or part of cells classified as S-phase) by comparing the relative coverage of the bin in *C* to its relative coverage in G1 cells:

$${{{{{\rm{s}}}}}}{{{{{\rm{core}}}}}}{{{{{{\rm{E}}}}}}}_{j}^{C}={{\log }}\left((\left|{G}_{1}\right|/\left|C\right|)\mathop{\sum}\limits_{\left\{i\in C\right\}}{{{{{\rm{ds}}}}}}{{{{{{\rm{n}}}}}}}_{j}^{i}/\mathop{\sum}\limits_{\left\{i\in {G}_{1}\right\}}{{{{{\rm{ds}}}}}}{{{{{{\rm{n}}}}}}}_{j}^{i}\right)$$

#### Mapping gene expression to genomic bins and scHi-C clusters

We used UCSC gene annotation to determine for each gene (as defined by the MARS-seq or 10X pipeline) a transcription start site (TSS) coordinate. Gene expression profiles were generated as the fraction of UMI per gene observed in scRNA-seq metacells or group of metacells^27^. Given an expression profile ${e}_{g}$ we defined a profile over genomic bins ${e}_{j}$ by taking the maximal expression of all genes mapping to TSSs on the bin *j*.

To match expression profiles and scHi-C clusters, we used clusters of TSSs showing coordinated or enriched expression to compute ${{{{{\rm{mean}}}}}}({{{{{{\rm{scoreA}}}}}}}_{\{j\in {{{{{\rm{TSSbins}}}}}}\}}^{C})$ or ${{{{{\rm{mean}}}}}}({{{{{{\rm{scoreE}}}}}}}_{\{j\in {{{{{\rm{TSSbins}}}}}}\}}^{C})$ for each scHi-C cluster C. ${{{{{\rm{TSSbins}}}}}}$ sets were generated in two ways. First, given a metacell model, we normalized expression (log transforming and subtracting the mean over all metacell profiles), and selected the top 50 enriched TSSs that had enrichment value larger than 0.5. These TSS sets were used to compute the matching between A and early scores and the erythrocyte scHi-C cluster in Fig. 1. Second, we clustered genes based on their metacell log2 UMI enrichment profiles (Fig. 3I), generating clusters that were curated manually and derived ${{{{{\rm{TSSbins}}}}}}$ sets from them for analysis of A-score and early score differences (Fig. 3J, K).

### Hi-C contact matrices analysis

#### Shaman analysis

To calculate enrichment of genome contacts in a Hi-C contact matrix, and to visualize chromosomal conformations, we used the Shaman algorithm^24,28^. We pooled all cells in each cell cluster, and down-sampled the contacts to the same number in each cell cluster pool. We then applied Shaman to the down-sampled contact pools. Briefly, Shaman shuffles contacts while maintaining the marginal coverage distribution and the contact distance distribution, creating a random shuffled contact matrix. The enrichment of a contact is then scored using a KS statistic on the k-nearest neighbors of that contact in the original down-sampled contact matrix and shuffled contact matrix. The Shaman results we report here were derived using an improved MCMC sampler that provide better convergence (in particular on matrices with a smaller number of contacts). In short, the algorithm uses efficient data structures to compute precisely the MCMC update rule. This approach is replacing the previously used strategy of adaptive calibration of a correction term for the function assigning probability for contacting at any genomic distance.

#### Insulation

We calculated insulation as described previously^24,29^. For a genomic locus, we counted the number of contacts where one contact is up to 200 kb upstream of the coordinate and the other up to 200 kb downstream. We next counted the number of contacts where both contacts are in distance up to 200 kb from the coordinate. The log ratio between these two numbers is the insulation score. We performed this calculation genome wide in 40 kb jumps.

#### Virtual 4 C

To calculate the 4 C trace at a specific genome coordinate *x*, we looked at all contacts which satisfy either of the next conditions:

- One of the fragment ends is at distance <3e3 from *x*, and the distance between the fragment ends is <1e5.
- One of the fragment ends is at distance <1e4 from *x*, and the distance between the fragment ends is between 1e5 and 5e5.
- One of the fragment ends is at distance <3e4 from *x*, and the distance between the fragment ends is between 5e5 and 1e6.

To screen for conformation differences for two scHi-C clusters in a set of target loci, we calculated the difference between their virtual 4 Cs. We partitioned the 4C trace to bins based on contact distance (2.5e4, 5e4, 1e5, 2e5, 3e5, 5e5, 7.5e5, 1e6, on both 3’ and 5’). We averaged the Shaman scores within every bin, and defined the distance of the conformations for two clusters as the maximum difference (in absolute value) over all bins.

### Parameters and specific figure panel analysis

#### Clustering ESC, Embryos and erythrocytes (Fig. 1)

We processed the scHi-C data, and performed QC and cell cycle phasing as described above. For generating clusters in Fig. 1, we used S-phase seeding (Supplementary Methods), with the following inputs: ${{K}^{S},K}^{{non}-S},\,{{{{{\rm{DSN}}}}}},$ and the matrices $c{A}_{j}^{i}$ and $c{B}_{j}^{i}$.

To identify primitive erythrocytes, we identified a bin cluster (of the 11 A-score-based bin clusters, see Supplementary Methods) that had high C3-specific A-score, and similarly a bin cluster with low C3-specific A-score. We calculated the pooled A-score of each of these two bin clusters in each single cell (denoted ${{{{{\rm{cell}}}}}}{\_}{{{{{{\rm{A}}}}}}}_{m}^{i}$ in the Supplementary Methods), and used a linear separator to classify cells based on these two scores as either C3 or non-C3 (Supplementary Fig. 4D). Embryo cells that were not classified as C3 were assigned to C2, and ESC cells to C1.

We generated the genomic bin expression value, A-score and early-score in ESC and non-pEry embryo as described above, in 40 kb resolution. We defined genomic bins with at least 4-fold change in expression as ESC- and embryo-induced. Similarly, we defined embryo A-specific bins and ESC A-specific bins as having at least 0.2 difference in A-score.

We screened for genes with different Shaman score in ESC and embryo using comparisons of virtual 4Cs as described above. We similarly calculated differences in Shaman scores for embryo and ESC A-specific bins (Supplementary Fig. 4A), but looking at the 4 C profile of each bin only up to 500 kb upstream and downstream.

#### Erythrocyte analysis (Fig. 2)

As before, we generated the genomic bin expression value, A-score and early-score in pEry and non-pEry in 40 kb resolution. We defined genomic bins with at least 4-fold change in expression as pEry- and non-pEry-induced. We also identified bins that were not expressed in either of the clusters.

To create Fig. 2J we identified 40 kb genomic bins with A-score that is at least 0.35 higher in Erys compared to embryo. We merged adjacent bins meeting this criterion, and for every set of merged bins found the bin with highest difference in A-score between Erys and embryo. For every such bin, we looked at the average A-score of its 3’ and 5’ bins up to 400 kb. We reversed A-score traces (mirroring 3’/5’) to create a matrix in which for all rows, the upstream 5’ A-score is higher. We concatenated the A-score trace in pErys and non-pErys, and clustered the concatenated traces using kmeans into 8 clusters. We performed a similar analysis when taking genomic bins with A-score that is at least 0.35 higher in embryo compared to Erys (Supplementary Fig. 4H).

To create Fig. 2L, we partitioned the contact matrix into 20 kb × 20 kb bins, and created for each of the loci in Fig. 2J a matrix of average Shaman scores in the 1 Mb around it (on both sides). We then averaged the scores in such matrices for the loci in each of the Fig. 2J clusters.

*Gata1 and Tal1 ChIP-seq*. We scored and normalized 20 bp bins for their *Gata1* and *Tal1* ChIP-seq score using data from ENCODE. We used ChIP-seq scores as previously described, computing ChIP coverage percentiles *p* for each bin, and defined the score as −log2(1 − *p*). We defined *Gata1* and *Tal1* binding sites as those having score > 8. For 40 kb genomic bins we computed a binding score as the maximum ChIP-seq score of all binding sites contained in it.

#### Clustering the embryo proper (Fig. 3)

We applied the replication trend mixture model (Supplementary Methods) on embryo scHi-C profiles classified as non-pEry, non-G1 and non-M as described above. We further selected cells with sufficient coverage (at least 8 contacts per 200 kb bin on average), and mid-S phase classification as in Nagano et al. 2017^19^.

We set ${n}_{{ij}}$ as the number of contacts in cell $i$ that map to genomic bin $j$ and excluded the X chromosome, or any bin with mean coverage <8. To set ${p}_{j}$, we calculated the fraction of contacts that mapped to each genomic bin across all G1 cells that are not erythrocytes.

To initialize the model we clustered cells hierarchically using distances based on correlations between rows in a normalized ${n}_{{ij}}$ matrix. Normalization provided initial heuristic correction to the cell cycle effect by ordering cells according to their scHi-C fraction of short range contacts and subtracting for each locus the running mean (using a window of 20 cells).

Given this clustering solution, we initialized $E\left[{z}_{{ik}}\right]$ such that each cell belongs to its cluster with probability 0.5, and to all other clusters with equal probability. In case $k=2$, each cell belongs to its cluster with probability $\frac{2}{3}$. To initialize ${s}_{i}$ we ordered the cells by the fraction of short-range contacts they make, and assigned them values between 1.2 and 1.8 according to their order, assuming that all parts of the replication program are equally represented in the data.

We performed cross validation on the hyperparameters, as described in the Supplementary Methods, and selected $L=12$, $R=11$, $\lambda=40$.

To generate UMAP projections of mid-S phase cells, we normalized ${n}_{{ij}}$ coverage by G1 mean coverage, selected bins with high variance to mean ratio, and calculated a cell-cell correlation matrix using these values. We then used the R package umap with default parameters (and random seed = 42). We repeated this analysis using data normalized based on inferred s-score (see Supplementary Methods).

##### Plotting replication trends for early replicating bins

To plot Fig. 3F, we identified bins that are in replication regime 2 (out of 12) in C2.1 (left plot; C2.3 for the right plot), and are in replication regime $\ge$4 in all other clusters, and for every cell calculated the total fraction of contacts from these bins.

##### Executing other scHi-C clustering algorithms

We executed schicluster and scHi-C topic modeling. We ran schicluster and topic modeling with resolutions 1 Mbp and 0.5 Mbp respectively, as performed in the publications of these methods.

##### Cluster annotation

To annotate the C2.1, C2.2 and C2.3 clusters, we used 15 TSS bin sets ${G}_{1},..{G}_{15}$ derived from the E9 metacell model data as described above. To account for possible differences in the s-score distribution in each cell cluster, we ordered genomic bins $j\in \mathop{\bigcup}\nolimits_{m}{G}_{m}$ by their mean early score across clusters, computed for each bin ${{{{{\rm{score}}}}}}{{{{{{\rm{E}}}}}}}_{j}^{{C}_{k}}$ and subtracted from it the running mean using a window of 200 bins, defining ${{{{{\rm{score}}}}}}{{{{{{{\rm{E}}}}}}}_{j}^{{\prime} }}^{{C}_{k}}$. We then computed ${{{{{\rm{mean}}}}}}({{{{{\rm{score}}}}}}{{{{{{{\rm{E}}}}}}}_{\left\{j\in {G}_{m}\right\}}^{{\prime} }}^{{C}_{k}})$, and normalized rows to create the matrix shown in Fig. 3J. A similar normalization strategy was used with A-scores to derive the matrix in 3 K. We repeated the same analysis for the gastrulation atlas metacell model, using 20 gene modules. We note that in order to test possible functional association of the C2.2 cluster, in this analysis we only used 42% of its cells showing a stronger correlation structure. Similar results were obtained using the entire C2.2 cluster.

To compare C2.1 and C2.3 to E14.5 data^24,33^, we computed A-scores for genomic bins of length 40Kbp in four samples: C2.1 cells, C2.3 cells, E14.5 HSCs and E14.5 NPCs. To compute these scores, we used the strict-early and strict-late genomic bins that we used to calculate A-scores previously. Because of the large difference in depth between our data and the E14.5 data, we downsampled the contacts of each genomic bin such that the total number of strict-early and strict-late contacts a genomic bin makes is the same in the four different samples. The downsampled contacts were used to calculate the A-scores.

To estimate our assay’s sensitivity, we sampled 100, 75, 50 and 25 cells from cluster C2.1, and applied the replication mixture model to a dataset including this subset with all cells from C2.2 and C2.3. We performed a similar analysis for C2.3.

To search for additional sub-structure in cluster C2.1 and C2.3 (Supplementary Fig. 9) we applied hierarchical clustering to cell-cell correlations derived using s-score normalized copy number profiles. We partitioned C2.1 into 3 subclusters, and C2.3 into 9 subclusters. To annotate the C2.1 subclusters, we correlated their A-score and coverage fold changes with differential gene expression of ectodermal cell types. To calculate the gene expression profile of a cell type, we calculated the average log2 expression of each gene across all the cell type’s metacells in the E9 scRNA-cell data. We then subtracted from each gene its mean expression across ectodermal cell types. This gave each gene its differential expression across all ectodermal cell types. To calculate a genomic bin’s A-score fold change in a subcluster, we calculated the A-score by pooling all contacts from the subcluster’s cells, and the A-score by pooling all contacts from the other subclusters’ cells. The bin’s A-score fold change is then the log2 of the ratio between these A-scores. To calculate the coverage fold change, we calculated the relative coverage in the pool of the subcluster’s cells as described above, and the relative coverage in the pool of other subclusters’ cells, and took the log2 of their ratio. We then only selected genes with at least 2-fold change in gene expression in some cell type, and correlated their relative expression with the A-score and coverage fold changes of the bins containing these genes. Both the A-score and coverage were calculated for genomic bins of size 200 kb. To annotate the C2.3 subclusters we did a similar analysis, but used only genes with at least 4-fold change in gene expression in some cell type.

#### Screening for differential ecto/meso contacts (Fig. 4)

We identified enhancers using Chip-seq ENCODE data from ectoderm (forebrain, midbrain, hindbrain) and mesoderm (heart and limb) tissues^43^. We calculated the ChIP-seq scores (log2(1-percentile)) in 20 bp resolution for each of the 5 tissues. We called enhancers as contiguous genomic intervals (or peaks) showing H3k4me1 scores > 7 (that is, the top 1/128 bins). We scored each peak H3k4me1 occupancy in mesoderm (maximum between the values of the two tissues) and ectoderm (maximum among the values of the three tissues). To define mesoderm and ectoderm specific peaks we required a score of at least 9 in one set of tissues and a difference of at least 3 between the scores of the two tissue sets. Overall this approached generated 24059 and 9506 meso and ecto- specific enhancers respectively.

To identify meso- and ecto- specifically expressed genes (and TSSs), we identified three metacells representing the mesoderm transcriptional state and three others representing the ectoderm state. We computed the maximum expression per gene in each set. 826 genes were showing at least two-fold difference between the two profiles, and their TSSs were considered for enhancer associations below.

To create a set of potential promoter-enhancer interactions, we linked each enhancer peak with its closest TSSs. We searched for such TSSs 50k-500k upstream and downstream of the enhancer locus (so up to two genes were linked with each peak). We did not use pairings spanning less than 50 kb since scHi-C resolution at such distances is limited. We also note that our pairing heuristic is by no means exhaustive, and is meant only to generate a shortlist of putative pairs.

Finally, we identified mesoderm and ectoderm specific enhancer-promoter candidate pairs as those involving a differential enhancer and a specifically expressed gene according to the definitions above. We defined the Shaman score of a putative enhancer-promoter interaction as the score of the contact between coordinates closest to the enhancer and promoter (using Euclidean distances)), and computed it for both meso and ecto. We selected pairs with Shaman score at least 15 higher in the expected cell cluster, or with an absolute shaman score at least 15 if the other score is negative, as having three way support.

To derive a *p*-value for the number of contacts between an enhancer and a promoter, we counted the number of contacts in the pooled ectoderm and mesoderm cells in a 50 kb window (25 kb to each direction) around the enhancer-promoter in the contact matrix. Denote these numbers for an enhancer-promoter pair *ep* by ${{{{{{\rm{ecto}}}}}}}_{{ep}}$ and ${{{{{{\rm{meso}}}}}}}_{{ep}}$. We similarly counted the number of contacts for all other enhancers and their associated TSSs. To test whether an enhancer-promoter pair had a high number of contacts in ectoderm, we calculated ${{{{{{\rm{ecto}}}}}}}_{{ep}}-\,{{{{{{\rm{meso}}}}}}}_{{ep}}$, and compared it to the empirical distribution of ${{{{{{\rm{ecto}}}}}}}_{{e{{\hbox{'}}}p{{\hbox{'}}}}}-\,{{{{{{\rm{meso}}}}}}}_{{e{{\hbox{'}}}p{{\hbox{'}}}}}$ for all background enhancer promoter pairs *e’p’* that had the same ${{{{{{\rm{ecto}}}}}}}_{{e{{\hbox{'}}}p{{\hbox{'}}}}}+\,{{{{{{\rm{meso}}}}}}}_{{e{{\hbox{'}}}p{{\hbox{'}}}}}$ value as *ep*. To increase power, for Supplementary Fig. 10C, D we only looked at enhancer-promoter pairs for which ${{{{{{\rm{ecto}}}}}}}_{{ep}}+\,{{{{{{\rm{meso}}}}}}}_{{ep}}\ge 80$. For the background distribution to be accurate, we only considered ${{{{{{\rm{ecto}}}}}}}_{{ep}}+\,{{{{{{\rm{meso}}}}}}}_{{ep}}$ values with more than 100 other enhancer-promoter pairs with similar ${{{{{{\rm{ecto}}}}}}}_{{e{{\hbox{'}}}p{{\hbox{'}}}}}+\,{{{{{{\rm{meso}}}}}}}_{{e{{\hbox{'}}}p{{\hbox{'}}}}}$ value. We performed a similar analysis for mesoderm.

We performed a similar analysis to identify H3k27me3-gene pairs. Genes were selected similarly. H3k27me3 regions were selected as those with ChIP-seq score > 7. We designated ecto- or mesoderm specific regions as those having ChIP-seq score > 3 higher than the other tissue. The selected pairs are those where the gene is lowly expressed and the H3k27me3 signal is higher.

To find all hotspots with support for different chromosomal conformation between ectoderm and mesoderm, we looked only at contacts in distance 1e4 to 1e6. We compared the Shaman score of every meso contact to the Shaman score of its nearest ecto contact. We detected regions with high Shaman difference iteratively. In each iteration we identified the contact with the maximal Shaman difference between meso and ecto, and removed all contacts where both their ends are in distance <5e4 from the maximal-difference contact. We continued with this process until no contact with Shaman difference >40 remained. This resulted in 5200 hits that we used in order to estimate the fraction of differential contacts explicable by known three-way supported promoter-enhancer pairing in the text.

#### Analysis of multiome-data and integration with pooled Hi-C clusters

We used scRNA-seq and scATAC-seq profiles from a recent paper by the Reik group^38^ to generate the analysis in Fig. 5, applying the following steps:

1. Using metacell-2^44^ with default parameters and target metacell size of 320 K UMIs to organize scRNA-seq profiles into 1404 metacells.
2. Using the RNA-based grouping of cells to collect single-cell ATAC reads and create a genomic track for each metacell.
3. Finding all genomic intervals with ATAC-coverage (total over all metacells) larger than 300 and identifying the maximal coverage 300 bp within each such interval as a peak. Overall this provided us with 94,600 peaks.
4. Grouping RNA metacells into 300 clusters using hierarchical clustering of the RNA signatures. RNA clusters were associated with cell type by comparison to gastrulation manifolds and TF expression profiles.
5. We then pooled ATAC reads over the clusters and extracted the reads within identified peaks into an accessibility count matrix. We removed cell clusters supported by fewer than 82 K ATAC reads, retaining for analysis 285 clusters.
6. Normalizing peak ATAC coverage in each cluster by normalizing (dividing by total reads for the cluster) and transforming the frequencies p to log2(1e-5+p).
7. Running kmeans++ with a large number of clusters (K = 120) over the normalized accessibility profiles. Deriving mean peak cluster profile by averaging the log normalized ATAC values.
8. Filtering peak clusters with less than 100 peaks (only 1 case). Annotating peak clusters as variable whenever at least four metacell clusters showed mean ATAC value smaller than −16 and the difference between minimum and maximum value over the cluster was larger than 0.7. All other clusters were considered constitutive.
9. Computing the A-score of each peak in ESC, Embryo, pEry, C2.1 and C2.3. Analysis of the A-score distributions in each cluster is used to generate Fig. 5B–D.
10. Identifying all pairs of peaks within less than 200 kb genomic distance. Summarizing the number of such pairs between elements of each pair of clusters into a matrix of observed “proximities”. Multiplying each element in the matrix by the total matrix counts divided by the product of its row and column total counts. Log transforming the resulted *enrichment ratio*, followed by hierarchical clustering of the submatrices defined by the constitutive and variable peak clusters (separately) in order to generate the heat map of Fig. 5E.

### Reporting summary

Further information on research design is available in the Nature Portfolio Reporting Summary linked to this article.

## Supplementary information

Supplementary information

Peer Review File

Description of Additional Supplementary Files

Supplementary Data 1

Supplementary Data 2

Supplementary Data 3

Supplementary Data 4

Reporting Summary

## Data Availability

The data that support this study are available from the corresponding authors upon reasonable request. The scHi-C and scRNA-seq data generated in this study have been deposited in the GEO database under accession code GSE148793. The ESC scHi-C data used in this study are available in the GEO database under accession code GSE94489. The previously published embryo gastrulation scRNA-seq data used in this study are available in the ArrayExpress database under accession code E-MTAB-6967. The scRNA/scATAC multiome data used in this study are available in the GEO database under accession code GSE205117. The neural progenitor cells’ Hi-C data used in this study are available in the GEO database under accession code GSE96107. The hematopoietic Hi-C data used in this study are available in the GEO database under accession code GSE119201.

## Code availability

All code supporting the analysis of this work is available in Github: https://github.com/tanaylab/scHiC_embryo.
