# Supplementary material for: Single cell Hi-C identifies plastic chromosome conformations underlying the gastrulation enhancer landscape
Source: Nat Commun. 2023 Jun 29;14:3844. doi: 10.1038/s41467-023-39549-4 (PMC10310791; doi:10.1038/s41467-023-39549-4)
Supplement: Supplementary file 1 — Supplementary information [file 41467_2023_39549_MOESM1_ESM.pdf]

Supplementary Information

Single cell Hi-C identifies plastic chromosome conformations underlying the gastrulation enhancer landscape

Nimrod Rappoport*[1,2], Elad Chomsky*[1], Takashi Nagano[3, 4], Charlie Seibert[5], Yaniv Lubling[1], Yael Baran[1], Aviezer Lifshitz[1], Wing Leung[3, 4], Zohar Mukamel[1], Ron Shamir[2], Peter Fraser[4, 5], Amos Tanay[1]

1 Department of Computer Science and Department of Biological Regulation, Weizmann Institute

2 The Blavatnik School of Computer Science, Tel Aviv University

3 Laboratory for Nuclear Dynamics, Institute for Protein Research, Osaka University

4 Nuclear Dynamics Programme, The Babraham Institute, Cambridge

5 Department of Biological Science, Florida State University

* Co-first authors

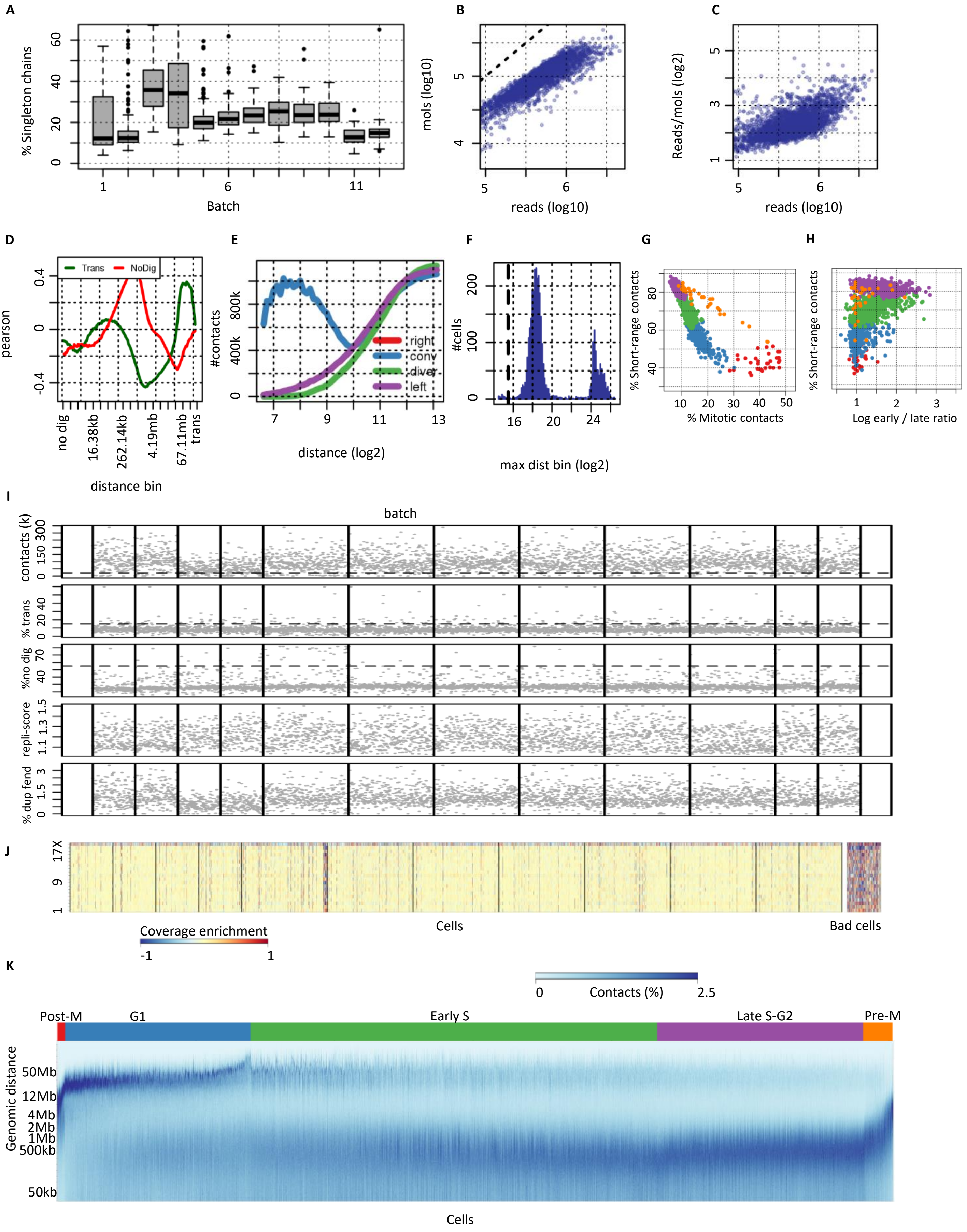
A
% Singleton chains
Batch
B
mols (log10)
reads (log10)
C
Reads/mols (log2)
reads (log10)
D
Trans
NoDig
pearson
no dig
16.38kb
262.14kb
4.19mb
67.11mb
trans
distance bin
E
#contacts
right
conv
diver
left
distance (log2)
F
#cells
max dist bin (log2)
G
% Short-range contacts
% Mitotic contacts
H
% Short-range contacts
Log early / late ratio
I
batch
contacts (k)
% trans
%no dig
repli-score
% dup fend
J
Coverage enrichment
Cells
Bad cells
K
Contacts (%)
Post-M
G1
Early S
Late S-G2
Pre-M
Genomic distance
50Mb
12Mb
4Mb
2Mb
1Mb
500kb
50kb
Cells

Supplementary Fig 1: Embryo scHi-C QC and cell cycle distributions

A: Fraction of singleton chains (reads with one restriction fragment) per batch. n equals 192 or 384 cells, depending on batch. Box limits are the first and third quartile, center line is the median, whiskers are 1.5 times the interquartile range, and points are outliers.

B-C: Shown are single cell read-depth vs number of ligation events extracted (B) or mean number of reads per molecule (C).

D: Correlation of the fraction of trans and non-digested contacts with the fraction of contacts in different contact distances.

E: Statistics on fragment end orientation at the ligation junction for reads coupling fragments within short genomic distance (from 128bp to 8192bp)

F: Distribution of the most frequent contact distance bin over all single cells.

G-H: Same as **Fig 1B-C**, using the mESC data. These panels are adapted from "Cell-cycle dynamics of chromosomal organization at single-cell resolution", Nagano et al., Nature 2017.

I: QC for single cells (dots) over 12 experimental batches.

J: Copy number enrichment score for chromosomes. Right cluster of scHi-C profiles represents highly aberrant profiles that were excluded from further analysis. Overall, we observed stable karyotypes in the embryo, as expected (and in contrast to ESCs)

K: Distribution of contact distance (color coded) for single cells (columns), ordered by inferred cell cycle stage (defined as in Nagano et al, 2017)

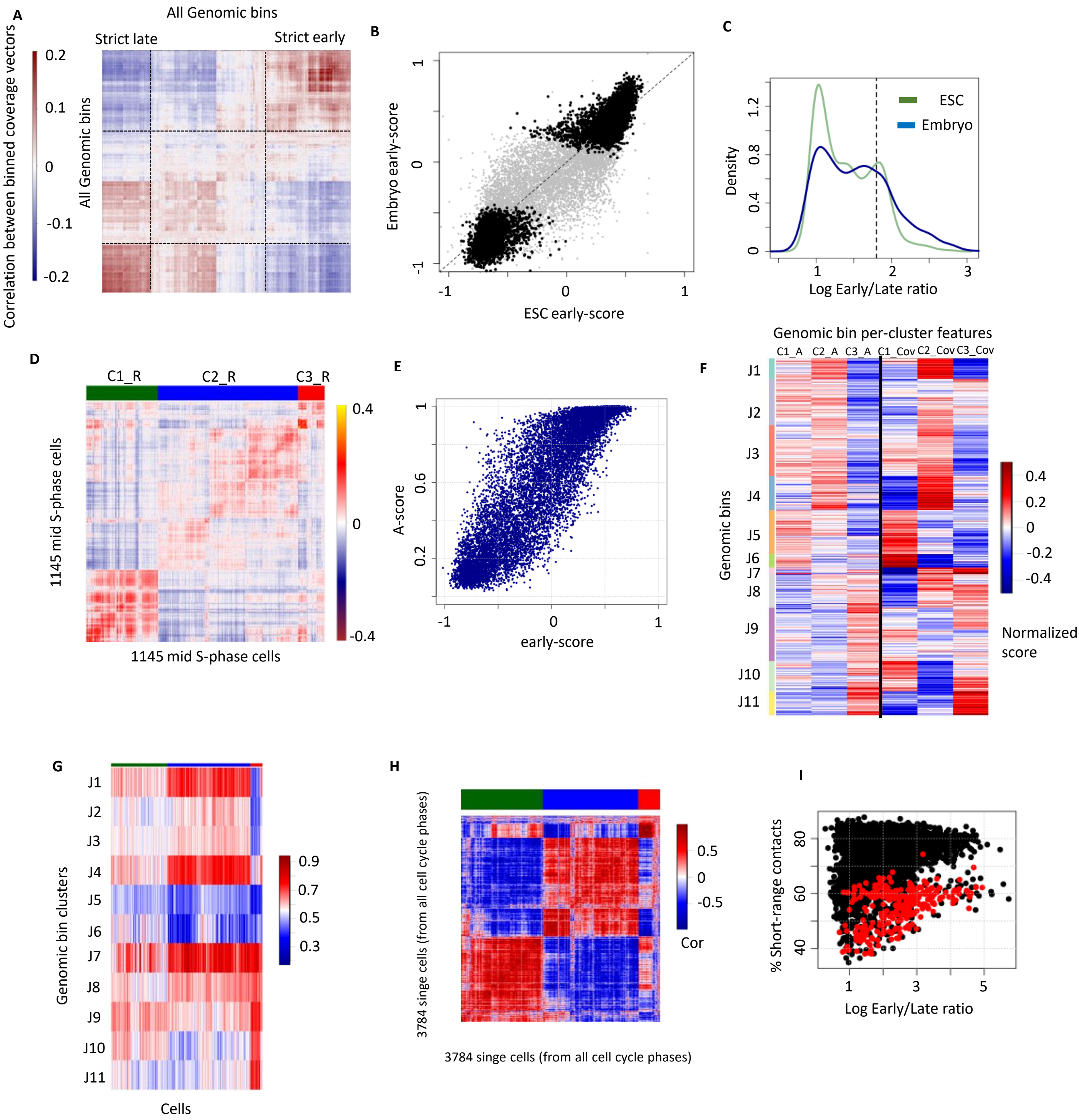

A
All Genomic bins
Strict late
Strict early
Correlation between binned coverage vectors
All Genomic bins
0.2
0.1
0
-0.1
-0.2
B
Embryo early-score
ESC early-score
C
ESC
Embryo
Density
Log Early/Late ratio
D
C1_R
C2_R
C3_R
1145 mid S-phase cells
1145 mid S-phase cells
0.4
0
-0.4
E
A-score
early-score
F
Genomic bin per-cluster features
C1_A
C2_A
C3_A
C1_Cov
C2_Cov
C3_Cov
Genomic bins
J1
J2
J3
J4
J5
J6
J7
J8
J9
J10
J11
Normalized score
G
Genomic bin clusters
Cells
H
3784 singe cells (from all cell cycle phases)
3784 singe cells (from all cell cycle phases)
Cor
I
% Short-range contacts
Log Early/Late ratio

Supplementary Fig 2: S-phase cluster seeding

A: Heatmap showing correlation of relative coverage profiles (number of contacts per 200kb genomic bin) over single ES and Embryo cells. Clustering was used to define two cohesive groups of bins that are used to define the “strict early” and “strict late” genomic signatures.

B: Each dot is a 200kb bin and the value for X and Y axes represent statistics for 344 ESC and 801 embryo cells, respectively. The values represent the log2 ratios of coverage for mid S-phase cells relative to cells in matching G1 cells. Black points represent bins in the strict early (top right) and strict late (bottom left) groups. These are showing consistent replication time behavior between ESC and Embryo cells.

C: Distribution of log2 single cell ratios between coverage on strict early and strict late genomic bins. These ratios serve as indicators for identifying replicating cells and are consistent between ESC (green) and embryo (blue) cells. The dashed line represents the threshold for defining mid-S phase cells in downstream analysis.

D: Heat map depicting cell-cycle normalized correlations between coverage profiles on 200kb bins for all mid-S phase cells (1145 in total). Hierarchical clustering was used to order profiles and define three coarse grained clusters, marked on top and labeled as C1_R, C2_R and C3_R.

E: Correspondence between early-score and A-score. For each 200kb genomic bin we compared the early-score (quantifying how early replicating is the bin on average) and the A-score (Quantifying how much the bin is contacting with strict-early bins).

F: Shown are clusters of genomic bins (rows, J1-J11), organized according to their normalized A-scores and early-scores in the three seed clusters (C1_R, C2_R, C3_R) (columns).

G: We calculate for every cell its normalized A-score for each of the 11 genomic bin groups shown in F. Cells are clustered into three groups (top bar) using this representation, extending the original C1_R, C2_R and C3_R seeds.

H: Correlation heat map of the clustering shown in G.

I: Comparing early / late ratio and percentage of short range contacts for all cells, where cluster C3 cells are marked in red.

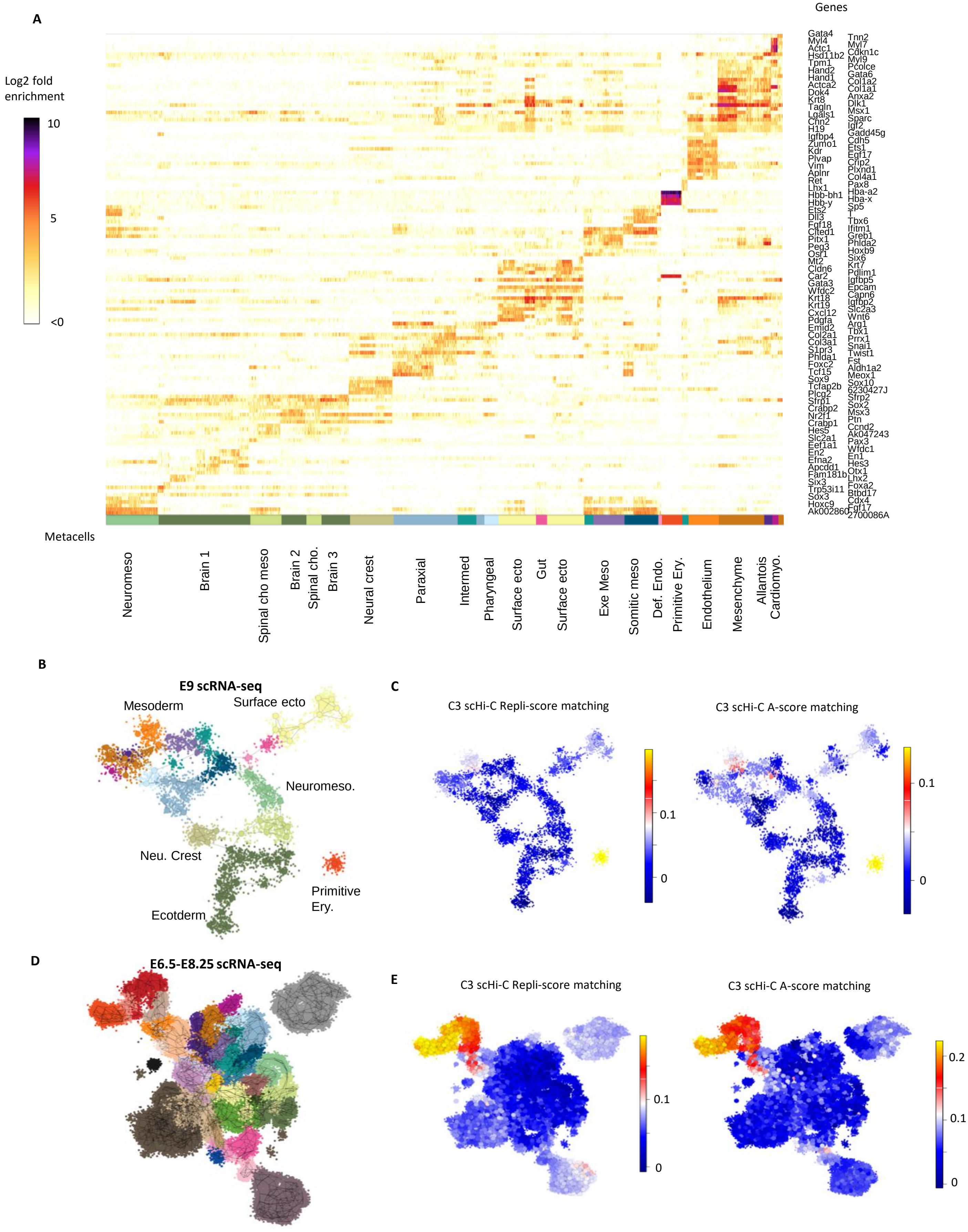
A
Log2 fold enrichment
10
5
<0
Genes
Gata4
Myl4
Actc1
Hsd11b2
Tpm1
Hand2
Hand1
Actca2
Dok4
Krt8
Tagln
Lgals1
Chn2
H19
Igfbp4
Zumo1
Kdr
Plvap
Vim
Aplnr
Ret
Lhx1
Hbb-bh1
Hbb-y
Ets2
Dll3
Fgf18
Cited1
Pitx1
Peg3
Osr1
Mt2
Cldn6
Car2
Gata3
Wfdc2
Krt18
Krt19
Cxcl12
Pdgfa
Emid2
Col2a1
Col3a1
S1pr3
Phlda1
Foxc2
Tcf15
Sox9
Tcfap2b
Plcg2
Sfrp1
Crabp2
Nr2f1
Crabp1
Hes5
Slc2a1
Eef1a1
En2
Efna2
Apcdd1
Fam181b
Six3
Trp53i11
Sox3
Hoxc9
Ak002860
Tnn2
Myl7
Cdkn1c
Myl9
Pcolce
Gata6
Col1a2
Col1a1
Anxa2
Dlk1
Msx1
Sparc
Igf2
Gadd45g
Cdh5
Ets1
Egfl7
Crip2
Plxnd1
Col4a1
Pax8
Hba-a2
Hba-x
Sp5
T
Tbx6
Ifitm1
Greb1
Phlda2
Hoxb9
Six6
Krt7
Pdlim1
Igfbp5
Epcam
Capn6
Igfbp2
Slc2a3
Wnt6
Arg1
Tbx1
Prrx1
Snai1
Twist1
Fst
Aldh1a2
Meox1
Sox10
6230427J
Sfrp2
Sox2
Msx3
Ptn
Ccnd2
Ak047243
Pax3
Wfdc1
En1
Hes3
Otx1
Lhx2
Foxa2
Btbd17
Cdx4
Fgf17
2700086A
Metacells
Neuromeso
Brain 1
Spinal cho meso
Brain 2
Spinal cho.
Brain 3
Neural crest
Paraxial
Intermed
Pharyngeal
Surface ecto
Gut
Surface ecto
Exe Meso
Somitic meso
Def. Endo.
Primitive Ery.
Endothelium
Mesenchyme
Allantois
Cardiomyo.
B
E9 scRNA-seq
Mesoderm
Surface ecto
Neuromeso.
Neu. Crest
Primitive Ery.
Ecotderm
C
C3 scHi-C Repli-score matching
0.1
0
C3 scHi-C A-score matching
0.1
0
D
E6.5-E8.25 scRNA-seq
E
C3 scHi-C Repli-score matching
0.1
0
C3 scHi-C A-score matching
0.2
0.1
0

Supplementary Fig 3: scRNA-seq reference map

A: Metacell model on 4712 single cell profiles acquired from E9.0 embryos. Marker genes (rows) are color coded by their expression in metacells. Metacell annotation is based on projection to the E6.5-E8.25 gastrulation atlas (bottom color bars).

B: Metacell 2d projection graph of the E9 scRNA-seq data.

C: Color coded maps showing matching between each metacell and the single cell Hi-C cluster 3, using early-score (left) or A-score (right) on regulated TSSs (methods).

D: Metacell projection for 110291 QC positive cells from the published scRNA-seq gastrulation atlas. Color coding is using majority voting on the original atlas annotation.

E: Similar to C, but using the gastrulation atlas.

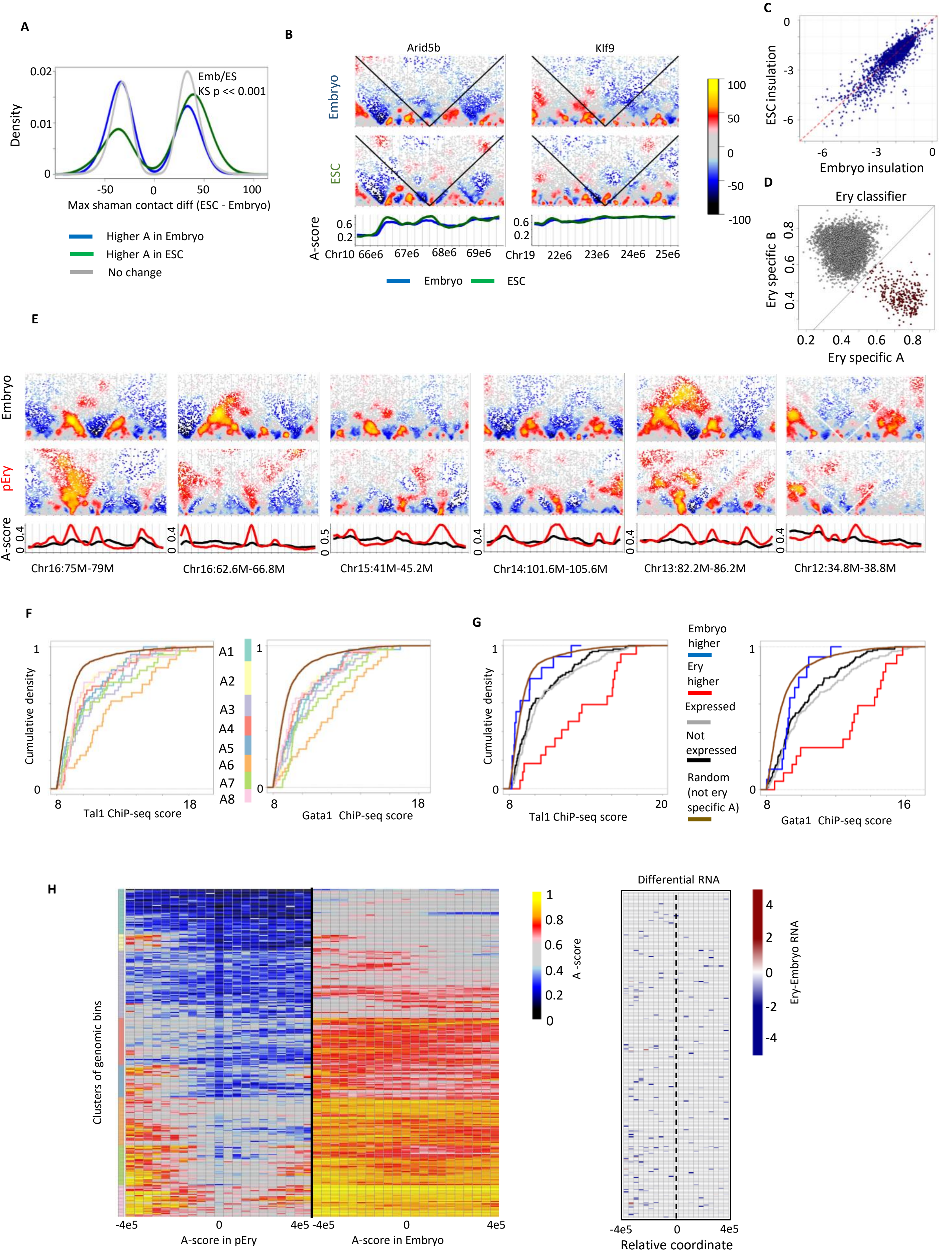

A
Emb/ES
KS p << 0.001
Density
Max shaman contact diff (ESC - Embryo)
Higher A in Embryo
Higher A in ESC
No change
B
Arid5b
Klf9
Embryo
ESC
A-score
Embryo
ESC
C
ESC insulation
Embryo insulation
D
Ery classifier
Ery specific B
Ery specific A
E
Embryo
pEry
A-score
Chr16:75M-79M
Chr16:62.6M-66.8M
Chr15:41M-45.2M
Chr14:101.6M-105.6M
Chr13:82.2M-86.2M
Chr12:34.8M-38.8M
F
Cumulative density
Tal1 ChIP-seq score
Gata1 ChIP-seq score
A1
A2
A3
A4
A5
A6
A7
A8
G
Embryo higher
Ery higher
Expressed
Not expressed
Random (not ery specific A)
Cumulative density
Tal1 ChIP-seq score
Gata1 ChIP-seq score
H
Clusters of genomic bins
A-score in pEry
A-score in Embryo
A -score
Differential RNA
Relative coordinate
Ery-Embryo RNA

Supplementary Fig 4: ESC, Embryo and pEry scHi-C conformation clusters

A: Embryo/ESCs differential contacts in loci grouped by differential A-score. We grouped 40kb genomic bins based on the differential A-score between ESC and non-pEry embryo cells. We created two virtual 4C profiles (from embryo and ESC pooled contacts) from the bin and up to 500kb upstream and downstream and identified the maximum difference between them (in absolute values). Positive values indicate higher contact enrichment in ESC and negative in non-pEry embryo cells. Shown are the distributions of the maximum 4C differences over bins in the three groups. The data are indicating ESC specific A-loci are significantly more engaged in differential contacts than Embryo specific A-loci (two-sided Kolmogorov–Smirnov test over n=600 ESC specific and n=554 Embryo specific A-loci).

B: Two examples of loci with differential contacts between ESC and embryo, but no significant change in A-score.

C: Comparison of contact insulation scores in the embryo and ESC.

D: Classification of pEry cells. We compared contacts in the C3 and C2 cell clusters to identify loci forming more contacts with strict-early bins in C3 as Ery-specific A bins, and similarly Ery-specific B bins. We then scored single cells using the ratio between Ery specific A bins and strict early/strict late bins generating the Ery specific A score (X axis), and similarly the Ery specific B score using the Ery-specific B bins (Y axis). Plotted are these two scores for all embryo cells, where the threshold used for classifying pEry cells is marked by a dashed line.

E: Examples of genomic domains with several Ery specific A-associated hotspots.

F: Cumulative distributions for *Tal1* and *Gata1* ChIP-seq values over loci in the Ery specific A clusters A1-A8 shown in **Fig 2**. Distribution over randomly sampled genomic bins is shown for reference in brown.

G: Cumulative distributions for *Tal1* and *Gata1* ChIP-seq values over loci in Ery specific A bins, classified according to transcriptional output from the bin in Ery and non Ery embryo metacells. Distribution over randomly sampled genomic bins is shown for reference in brown.

H: Similar to **Fig 2J,K**, but clustering data from bins showing Embryo specific high A-scores.

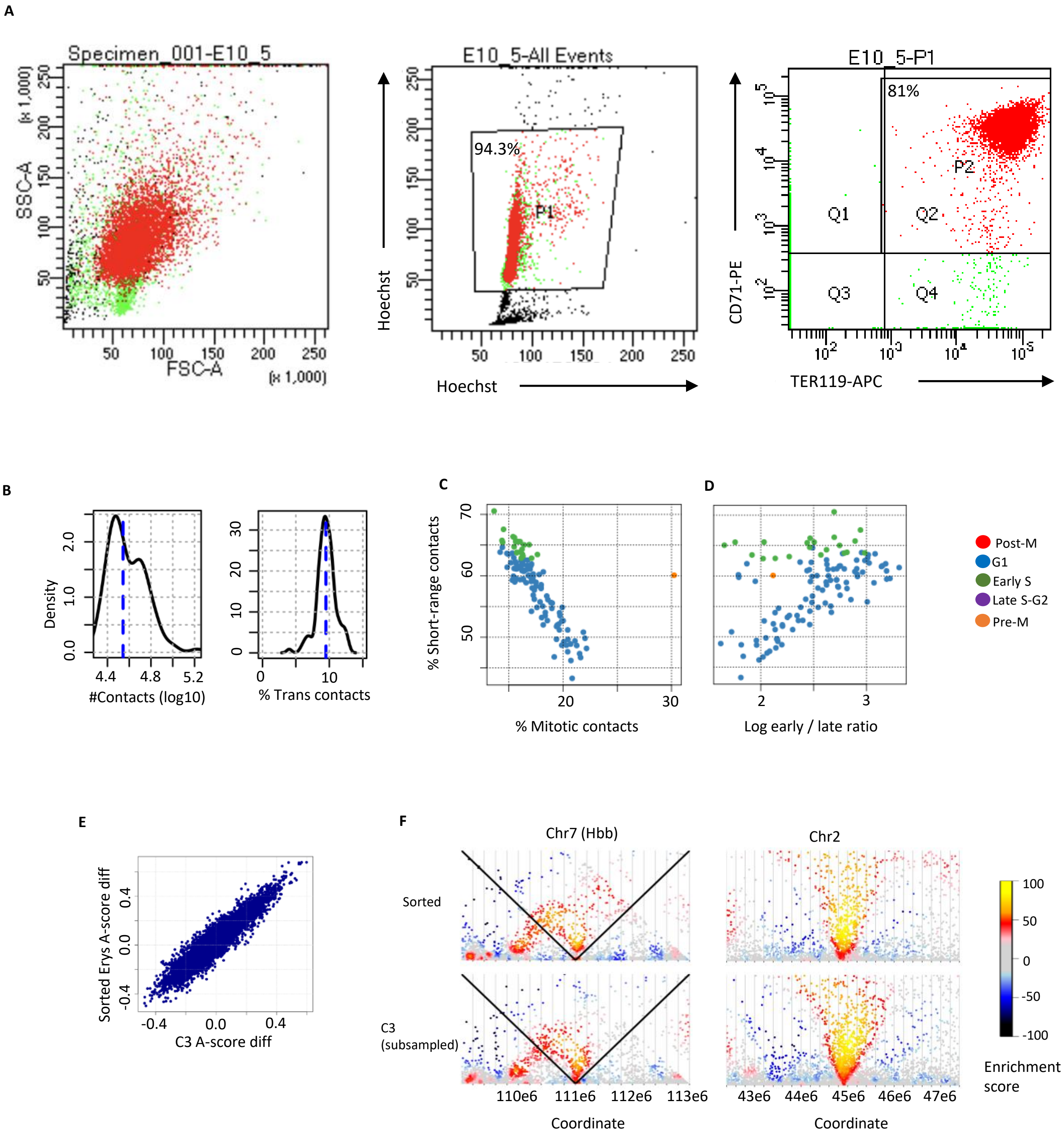

A
Specimen_001-E10_5
SSC-A (x 1,000)
FSC-A (x 1,000)
E10_5-All Events
94.3%
P1
Hoechst
Hoechst
E10_5-P1
81%
P2
Q1
Q2
Q3
Q4
CD71-PE
TER119-APC
B
Density
#Contacts (log10)
% Trans contacts
C
% Short-range contacts
% Mitotic contacts
D
Log early / late ratio
Post-M
G1
Early S
Late S-G2
Pre-M
E
Sorted Erys A-score diff
C3 A-score diff
F
Chr7 (Hbb)
Chr2
Sorted
C3
(subsampled)
110e6
111e6
112e6
113e6
43e6
44e6
45e6
46e6
47e6
Coordinate
Coordinate
Enrichment
score

Supplementary Fig 5: scHi-C in sorted primitive erythrocyte

A: Sorting of Ter119+/CD71+ primitive erythrocytes.

B: Distributions of coverage and percent of trans-chromosomal contacts for scHi-C data derived from 118 sorted primitive erythrocytes.

C-D: Similar to **Fig 1B-C** but showing data on sorted pEry cells. Note the different range in the Y-axis, as all analyzed pErys show high level of long-range contacts (also causing their G1 annotation using the default cell cycle phasing algorithm).

E: Comparison of the difference between A-score in pEry cells sorted in-silico to embryo A-scores (X), and difference between A-scores pEry cells sorted in-vivo to embryo A-scores (Y) for 40kb genomic bins.

F: Comparing contact maps derived from pEry cells (clustered and sorted) in two loci.

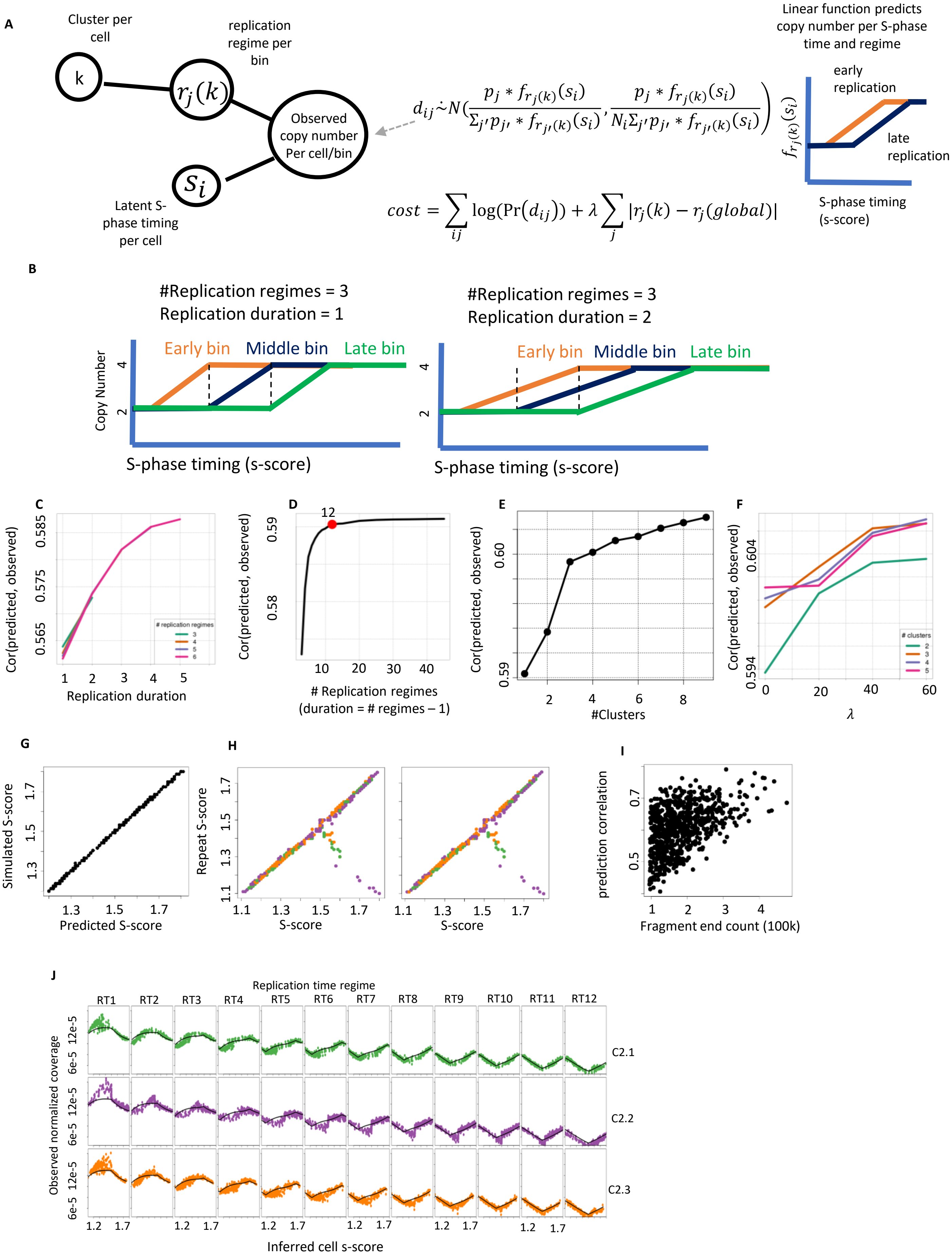
A
Cluster per cell
replication regime per bin
Observed copy number Per cell/bin
Latent S-phase timing per cell
Linear function predicts copy number per S-phase time and regime
early replication
late replication
S-phase timing (s-score)
B
#Replication regimes = 3
Replication duration = 1
#Replication regimes = 3
Replication duration = 2
Early bin
Middle bin
Late bin
Copy Number
S-phase timing (s-score)
C
Cor(predicted, observed)
Replication duration
replication regimes
D
Replication regimes
(duration = # regimes – 1)
E
#Clusters
F
clusters
G
Simulated S-score
Predicted S-score
H
Repeat S-score
S-score
I
prediction correlation
Fragment end count (100k)
J
Replication time regime
RT1
RT2
RT3
RT4
RT5
RT6
RT7
RT8
RT9
RT10
RT11
RT12
C2.1
C2.2
C2.3
Observed normalized coverage
Inferred cell s-score

Supplementary Fig 6: Parametric mixture model for clustering scHi-C replication profiles

A: Model’s schematic. We assign each replicating single cell with two latent variables, one representing its replication time and the other associating it with a cluster. The model defines the distribution of contacts per genomic bin based on these two variables, and using cluster specific association between genomic bins and replication time regime (represented on the right). We infer model parameters and latent variable association by optimizing a likelihood function with an additional regularization component.

B: We define a set of replication regimes assuming constant replication rates (linear increase per time) and a duration parameter defining the degree of overlap between replication regimes.

C: We used cross validation with the embryo scHi-C data to select model parameters, here showing the effect of the replication duration parameter on the predictive value, using just one cluster.

D: Effect of the number of distinct replication regimes on model performance in cross validation, using one cluster. Red dot signifies the selected value.

E: Effect of the number of clusters on model performance.

F: Effect of the regularization parameter lambda on model performance.

G: We simulated single cell profiles and inferred their S-phase timing variable. Shown here is the correspondence between simulated and inferred values.

H: We tested the robustness of our inference approach by clustering a subsampled dataset. Cells are colored by their original clustering (left), and clustering in the subsample (right). We observed a small number of cells with mirror S-phase time values. Even for these cells the cluster association was stable.

I: For each genomic bin, we used cross validation to compare predicted and observed genomic coverage over all cells in the model. Bins with high coverage (X-axis) provide better predictive power.

J: Shown are data for non-pEry cells in three clusters (rows) across 12 replication regimes (columns), where in each panel points represent cells. The cell’s inferred s-score (X) and total normalized coverage (Y) for genomic bins associated with the column’s replication regime in the cell cluster are shown. Each row shows data only

for cells that belong to the respective cluster. Black lines represent the model's predicted normalized coverage.

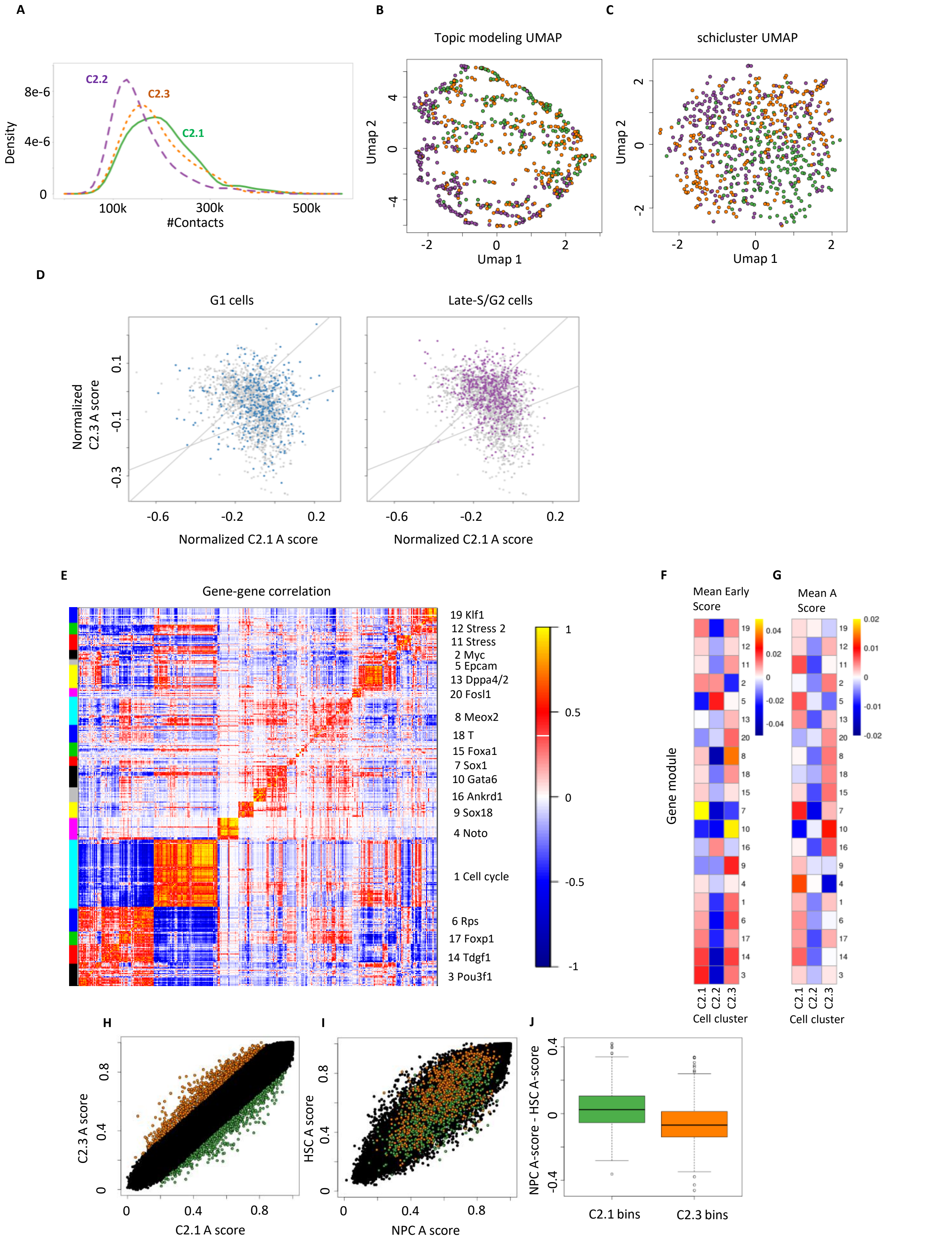
A
C2.2
C2.3
C2.1
Density
8e-6
4e-6
0
100k
300k
500k
#Contacts
B
Topic modeling UMAP
Umap 2
Umap 1
C
schicluster UMAP
Umap 2
Umap 1
D
G1 cells
Late-S/G2 cells
Normalized C2.3 A score
Normalized C2.1 A score
E
Gene-gene correlation
19 Klf1
12 Stress 2
11 Stress
2 Myc
5 Epcam
13 Dppa4/2
20 Fosl1
8 Meox2
18 T
15 Foxa1
7 Sox1
10 Gata6
16 Ankrd1
9 Sox18
4 Noto
1 Cell cycle
6 Rps
17 Foxp1
14 Tdgf1
3 Pou3f1
F
Mean Early Score
G
Mean A Score
Gene module
C2.1
C2.2
C2.3
Cell cluster
H
C2.3 A score
C2.1 A score
I
HSC A score
NPC A score
J
NPC A-score - HSC A-score
C2.1 bins
C2.3 bins

Supplementary Fig 7: **Annotating and validating embryo scHi-C clustering**

A: Distribution of the number of contacts in cells of the three embryo clusters.

B-C: UMAP projection of the low dimensional representation obtained using a topic modeling approach and schicluster for cells from the three embryo-proper clusters.
D: Similar to **Fig 3H**, but projecting G1 cells (left) or late S/G2 cells (right), demonstrating poor separation of G1 cells.

E-G: Similar to **Fig 3I-K** but using gene modules inferred from the gastrulation E6.5-E8.25 atlas.

H: A-score for genomic bins in C2.1 (X) and C2.3 (Y) cells. Bins with differential C2.1 and C2.3 A-scores are highlighted.

I: A-score for genomic bins in E14.5 NPC (X) and HSC (Y). Genomic bins are colored as in H.

J: The difference in A-score between NPC and HSC for genomic bins with high C2.1 A-score (n=651, left) and high C2.3 A-score (n=863, right). Box limits are the first and third quartile, center line is the median, whiskers are 1.5 times the interquartile range, and points are outliers.

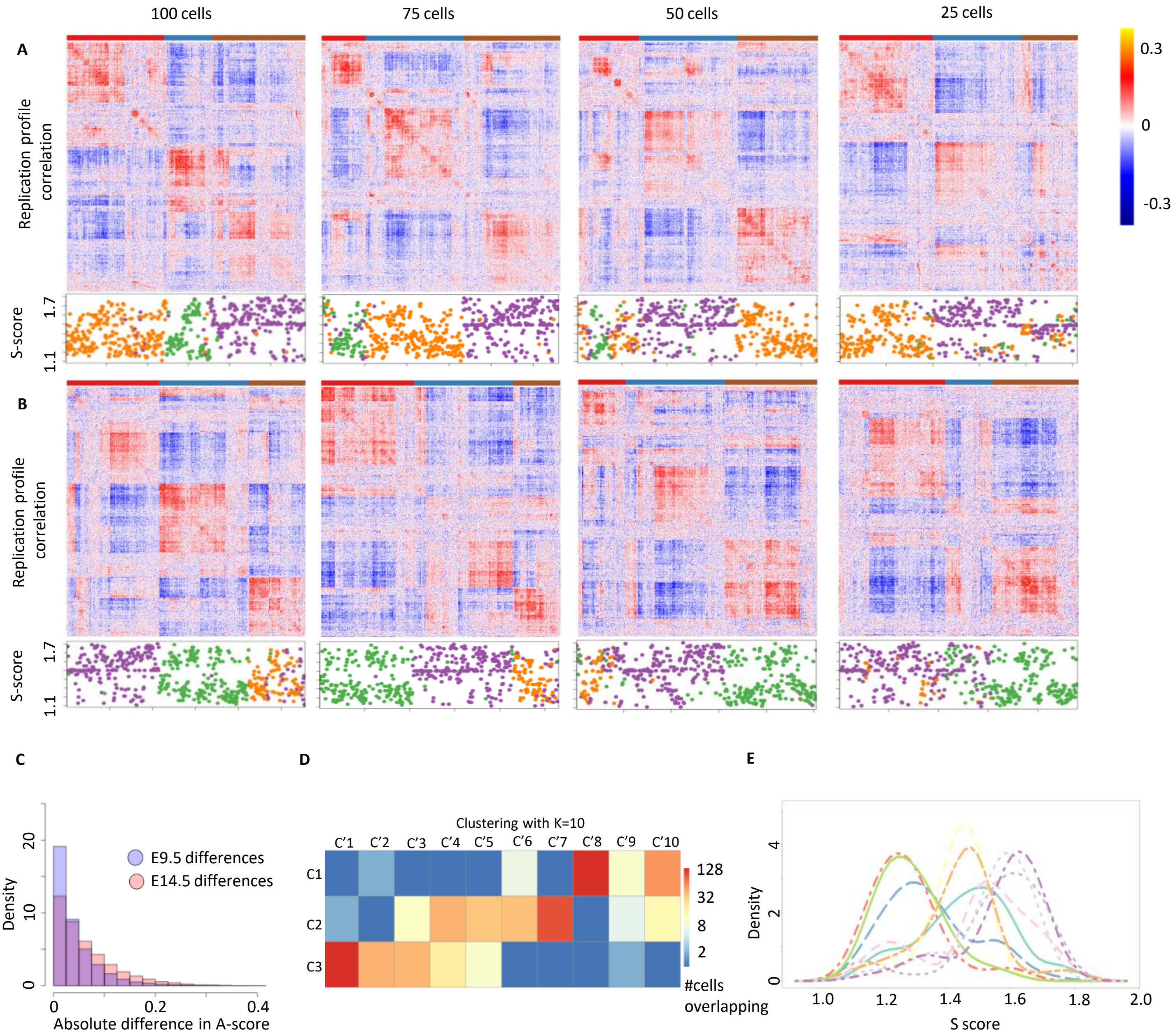
100 cells
75 cells
50 cells
25 cells
A
B
C
D
E
Replication profile correlation
S-score
1.7
1.1
0.3
0
-0.3
Density
E9.5 differences
E14.5 differences
Absolute difference in A-score
Clustering with K=10
C'1
C'2
C'3
C'4
C'5
C'6
C'7
C'8
C'9
C'10
C1
C2
C3
128
32
8
2
#cells overlapping
S score

Supplementary Fig 8: Sensitivity analysis

A: We sampled 100, 75, 50 and 25 cells from C2.1, and reran the model with these cells and all cells from C2.2 and C2.3. For every run we plot the correlation structure between coverage profiles of cells after normalizing the cell cycle effect (top), and the inferred S-phase (bottom). Inferred S-phase is colored by the original cell clustering, and the coverage correlations are annotated by the newly detected clusters (top bar).

B: Similar to A, for C2.3 cluster.

C: Distribution of the absolute differences in A-score between E9.5 C2.1 and C2.3, and E14.5 NPC and HSC.

D: Comparing the results of the algorithm using 10 clusters to the inferred 3 cluster structure, showing for each small cluster (columns) the distribution of number of cells in big clusters.

E: Distribution of inferred s-score for the 10 clusters in D. Data indicate that the replication signal is dominating the cluster structure when going beyond three clusters.

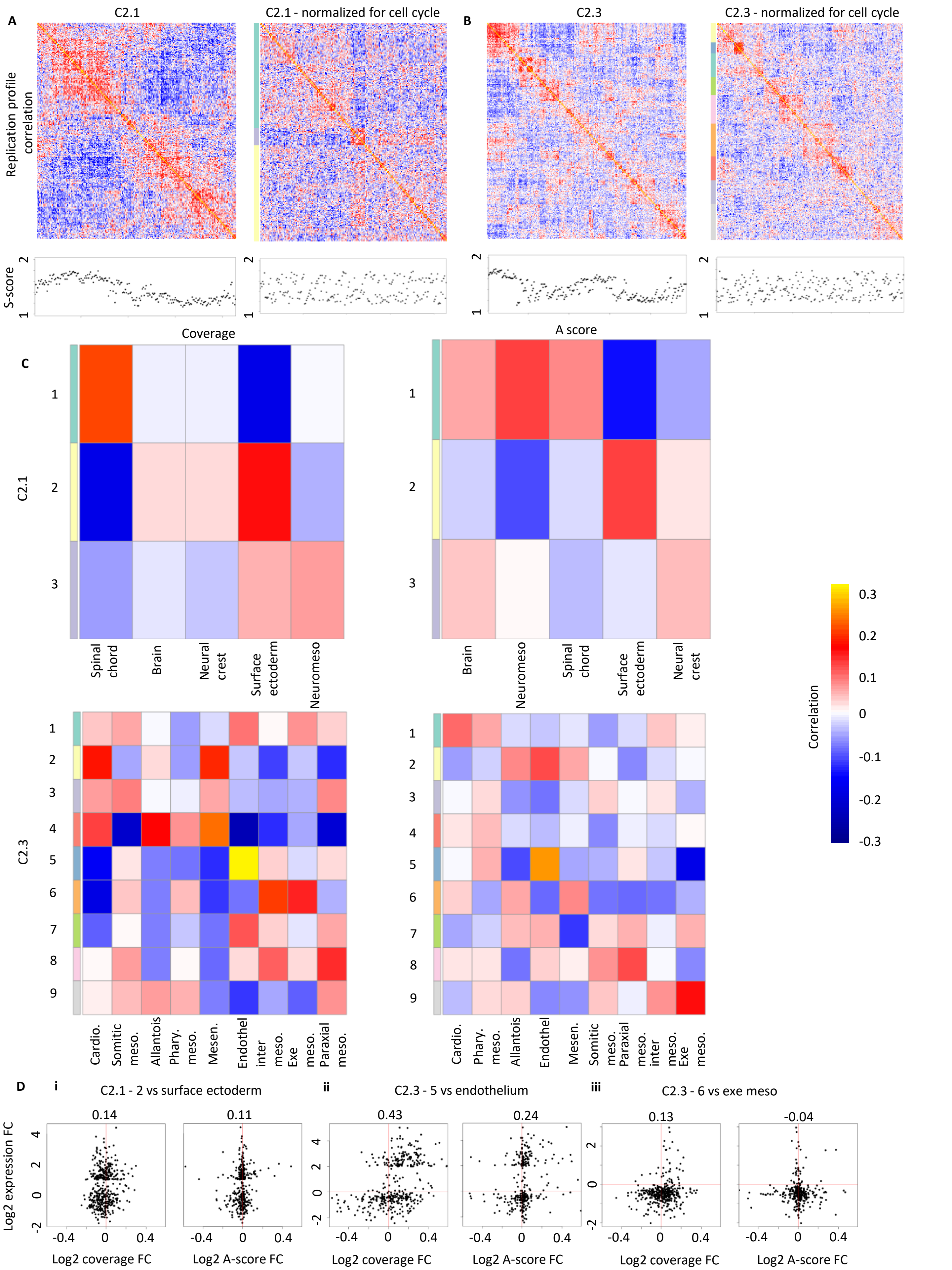

A
C2.1
C2.1 - normalized for cell cycle
B
C2.3
C2.3 - normalized for cell cycle
Replication profile correlation
S-score
Coverage
A score
C
C2.1
C2.3
Spinal chord
Brain
Neural crest
Surface ectoderm
Neuromeso
Cardio.
Somitic meso.
Allantois
Phary. meso.
Mesen.
Endothel
inter meso.
Exe meso.
Paraxial meso.
Correlation
D
i
C2.1 - 2 vs surface ectoderm
ii
C2.3 - 5 vs endothelium
iii
C2.3 - 6 vs exe meso
0.14
0.11
0.43
0.24
0.13
-0.04
Log2 expression FC
Log2 coverage FC
Log2 A-score FC

Supplementary Fig 9: Further subpartitioning of the scHi-C clusters

A: Left: Showing the correlation structure among coverage profiles of cells in cluster C2.1, with inferred S-phase time depicted below. Most structure in the non-normalized profile stems from the replication cycle. Right: Similar to A, but using coverage profiles that were normalized by the inferred s-score.

B: Same as A, for the C2.3 cluster.

C: Top left: The correlation between differential coverage of C2.1 subclusters (rows) and differential expression of ectodermal cell types (columns). Top right: same as top left, but the correlation is between differential A-score and differential expression. Bottom panels are as the top panels, but using C2.3 subclusters and mesodermal cell types.

D: Each pair of panels shows the differential coverage (left, X) and A-score (right, X) of one scHi-C subcluster vs differential expression (Y) for one scRNA-seq cell type.

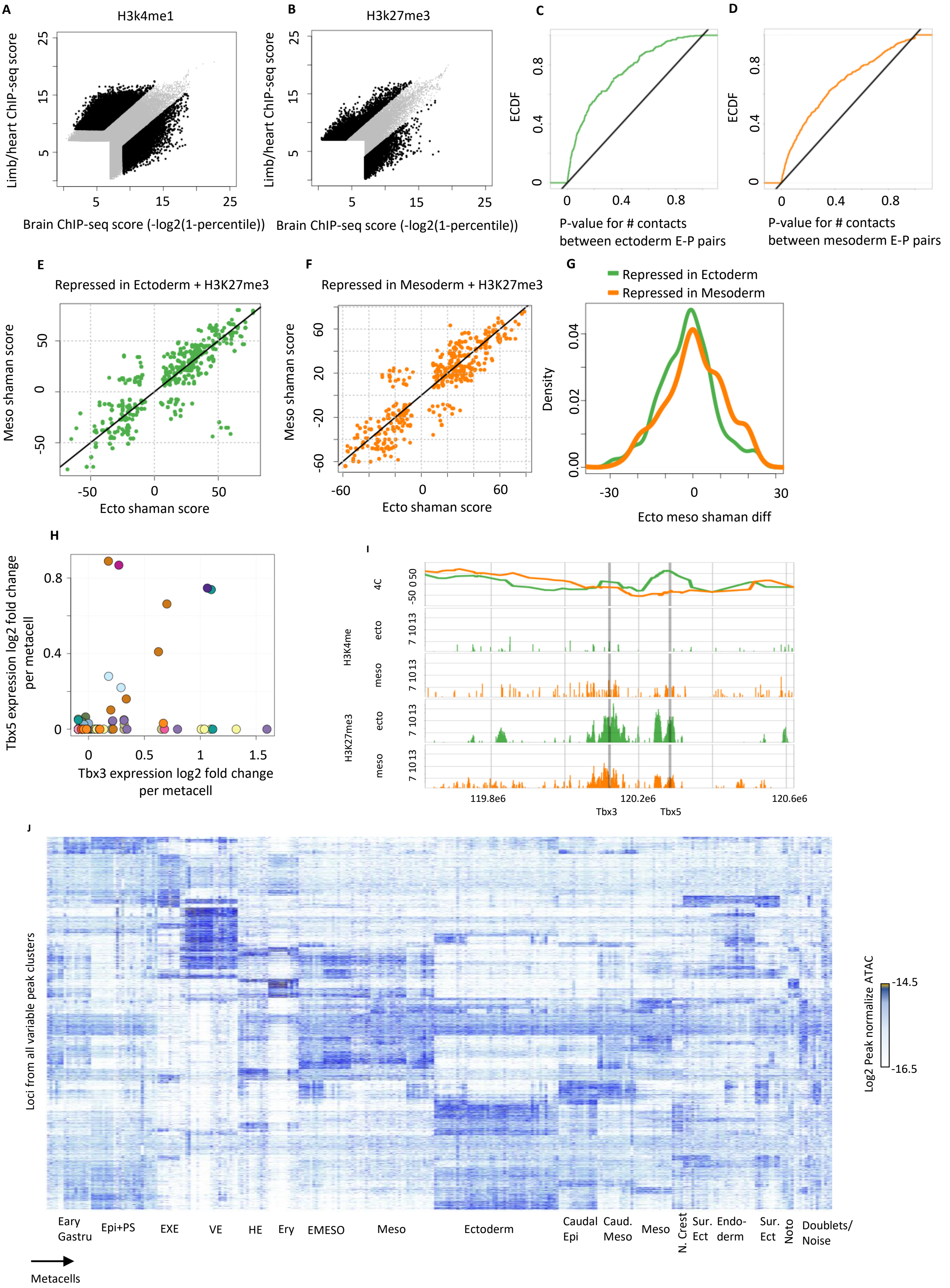
A
H3k4me1
Limb/heart ChIP-seq score
Brain ChIP-seq score (-log2(1-percentile))
B
H3k27me3
Limb/heart ChIP-seq score
Brain ChIP-seq score (-log2(1-percentile))
C
ECDF
P-value for # contacts
between ectoderm E-P pairs
D
ECDF
P-value for # contacts
between mesoderm E-P pairs
E
Repressed in Ectoderm + H3K27me3
Meso shaman score
Ecto shaman score
F
Repressed in Mesoderm + H3K27me3
Meso shaman score
Ecto shaman score
G
Repressed in Ectoderm
Repressed in Mesoderm
Density
Ecto meso shaman diff
H
Tbx5 expression log2 fold change per metacell
Tbx3 expression log2 fold change per metacell
I
4C
H3K4me
ecto
meso
H3K27me3
ecto
meso
119.8e6
120.2e6
120.6e6
Tbx3
Tbx5
J
Loci from all variable peak clusters
Log2 Peak normalize ATAC
-14.5
-16.5
Eary Gastru
Epi+PS
EXE
VE
HE
Ery
EMESO
Meso
Ectoderm
Caudal Epi
Caud. Meso
Meso
N. Crest
Sur. Ect
Endo-derm
Sur. Ect
Noto
Doublets/ Noise
Metacells

Supplementary Fig 10: Promoter-enhancer contacts

A-B: Defining putative ectoderm and mesoderm specific H3K4me1 and H3K27me3 hotspots. ChIP-seq data from encode (Methods) is normalized as log2(1-percentile). Threshold is depicted using black dots. Ectoderm scores are calculated using hind-, mid- and forebrain, and mesoderm using heart and limb.

C: We tested for an increased number of contacts in C2.1 cells compared to C2.3 cells in ectoderm enhancer-promoter pairs. ECDF of the p-values is shown. See Methods for the statistical test used.

D: Similar to C, but for mesoderm enhancer-promoter pairs.

E: Comparing Ecto and Meso Shaman scores on 199 pairs of ectoderm repressed TSSs and ectoderm specific H3k27me3 sites.

F: Similar to E, but for 154 mesoderm specific pairs.

G: Cumulative Shaman difference between ectoderm and mesoderm for the data in E-F.

H: Log2 fold change of *Tbx3* and *Tbx5* expression in the E9 metacell model.

I: Epigenomic landscape around the *Tbx3-Tbx5* (marked by gray lines) in ectoderm and mesoderm tissues.

J: Similar to 5A lower panel, but showing all peaks from all peak clusters (but excluding clusters with constitutively high accessibility over all metacells).

Supplementary Methods - Algorithms for clustering scHi-C data

1. Clustering I: the S-phase seeding algorithm

We describe an approach we term "S-phase seeding" to cluster scHi-C profiles. The algorithm works in three steps: i) We cluster only cells in K^S (mid S-phase cells) using normalized correlation between coverage profiles. ii) Using these "seed" cell clusters we partition the genome into bin clusters showing specific combinatorial compartment association between the seeds. iii) We score single cells from all cell cycle phases for the compartment association of the bin clusters, and cluster cells using these scores.

I. Algorithm's input: the input for the algorithm consists of the following:

a) *Early/late ratios* el^i for each cell i as defined previously, and the two resulted sets K^S, K^{non-S} defined using a threshold on the ratios.

b) *coverage profile* dsn_j^i : we first have to control the effect of uneven whole-chromosome coverage in scHi-C data (for cases that are not filtered in the pre-processing pipeline as QC negative). We search for cells *i* for which the coverage of some chromosome is at least 30% higher than the median over other cells. That is, $\Sigma_{j\in\mathrm{chrom}}\mathrm{dsn}_j^i > 1.3 * \mathrm{median}_{\mathrm{i}'}(\Sigma_{j\in\mathrm{chrom}}\mathrm{dsn}_j^{i'})$. We mask the dsn_j^i coverage profiles of such chromosomes (only in the affected cell) with NA values. In what follows we shall assume coverage statistics is masked.

c) A/B-association *profiles*. For each genomic bin j and each cell i, we count the number of long range intra-chromosomal contacts (>1Mb) observed between fragment ends in bins j and fragment ends in the $\mathrm{early}^{\mathrm{strict}}$ and $\mathrm{late}^{\mathrm{strict}}$ genome compartments, defining count vectors cA_j^i and cB_j^i.

II. Algorithm's stages:

Step I: In this step we cluster K^S cells using dsn_j^i coverage data. We first compute raw correlations $cor(\mathrm{dsn}^i, \mathrm{dsn}^{i'})$. The resulted correlation matrix is highly biased by the timing of cells within S-phase (even though we analyze only mid-S phase cells). For initial control over this effect, we order cells by their el^i values and normalize each row in the correlation matrix by subtracting from it a running median (using a window of 101 cells). We symmetrize the normalized correlation matrix and use it to cluster cells hierarchically into L clusters. At the end of this first step, a set of S-phase clusters are

defined as our *clustering seeds* $\mathrm{CS}_1, .. \mathrm{CS}_L$. See **Supplementary Fig 2D** for the results derived by applying this approach to embryo and ESC cells.

Step 2: In this step we use cell cluster seeds to group genomic bins into clusters with common compartment association over the seeds. We define the cluster coverage for bin j in cluster seed c as:

$$\mathrm{clscov}_j^c = \frac{\Sigma_{i\in c}\mathrm{dsn}_j^i}{\Sigma_{\mathrm{j}'}\Sigma_{\,i\in c}\mathrm{dsn}_{j'}^i}.$$

And the cluster enrichment ratio for a bin j is set to:

$$\mathrm{clsecov}_j^c = \log2\left(\mathrm{clscov}_j^c\right) - \log2(\mathrm{mean}_{c'}\left(\mathrm{clscov}_j^{c'}\right))$$

We also define the A-compartment association of a bin j for a given cluster c as:

$$\mathrm{clsA}_j^c = \sum_{\{i\in c\}} cA_j^i \,/ \left[\sum_{\{i\in c\}} cA_j^i + \sum_{\{i\in c\}} cB_j^i\right]$$

We filter bins showing low variation ($\max_c \mathrm{clsA}_j^c \leq 0.1$) and normalize:

$$\mathrm{clsA}_j^c = \mathrm{clsA}_j^c - \mathrm{mean}_{c'}(\mathrm{clsA}_j^{c'}).$$

Each genomic bin can now be defined by $2L$ features (L being the number of seed clusters) – L features from $\mathrm{clsecov}_j^c$ and L from clsA_j^c. We apply k-means to this representation and derive coarse grain partition of the genome into M *bin clusters*, $J_1, .., J_M$. **Supplementary Fig 2F** shows the genomic bin clustering as applied for data on ESC and embryo mid-S cells.

Step 3: In this step we use the genomic bin clusters inferred in step 2 to score and cluster single cells from all cell cycle phases. This is done by computing for each cell and bin cluster an A-association score:

$$\mathrm{cell\_A}_m^i = \sum_{\{j\in J_m\}} cA_j^i \,/ \left[\sum_{\{j\in J_m\}} cA_j^i + \sum_{\{j\in J_m\}} cB_j^i\right]$$

This gives rise to a vector of length *M* for each cell. These vectors are used to compute correlations between cells, and these correlations are clustered with hierarchical clustering. **Supplementary Fig 2G-H** demonstrate this analysis for the Embryo + ESC datasets**.**

III. *Limitations*. At the end of these three steps we define singe cell clusters based on the $\mathrm{cell\_A}_m^i$ features. Importantly, the algorithm can effectively identify clusters only for

proliferating cell types (as it seeds clusters on S-phase cells), even though the clusters will eventually involve cells across all cell cycle phases. In scenarios involving very homogeneous cell populations, the technique for seeding as described in step 1 may not be sensitive enough.

2. Clustering II: single cell *replication trend mixture modelling*

We next describe *replication trends mixture modelling*, a more sensitive approach for clustering scHi-C profiles that we used to dissect embryo cells, when the S-phase seeding algorithm that we described above failed to detect clusters due to lingering cell cycle variation effects. The mixture algorithm is defining a parametric model for DNA replication, describing how the copy number of genomic bins changes during S-phase in a cluster-specific fashion.

The input to the model is a matrix $\mathrm{Cov} = [n_{ij}]$ on genomic bins $j \in J$ and cells $i \in I$. n_{ij} is the number of contacts in cell i mapped to bin j. We define a generative model for this matrix as follows. Each genomic bin j is characterized by a probability p_j that a contact will be sampled from it for cells with uniform copy number (2 for all autosomes). For replicating cells, each genomic bin j and cell i is linked with a latent copy number c_{ij} which is a continuous number between 2 and 4. Denote by N_i the library size of cell i; that is, $N_i = \Sigma_j n_{ij}$. We assume that contacts are sampled from cell i by a multinomial distribution, such that n_{ij} is sampled with probability $\frac{p_j * c_{ij}}{\Sigma_{j'} p_{j'} * c_{ij'}}$. We use the normal approximation to the binomial distribution, and since $\frac{p_j * c_{ij}}{\Sigma_{j'} p_{j'} * c_{ij'}}$ is small, we assume that the mean and variance of the binomial distribution are equal, and therefore $n_{ij} \dot{\sim} N(\frac{p_j * c_{ij}}{\Sigma_{j'} p_{j'} * c_{ij'}} * N_i, \frac{p_j * c_{ij}}{\Sigma_{j'} p_{j'} * c_{ij'}} * N_i)$. Equivalently, denote by d_{ij} the cell-normalized coverage, $d_{ij} = n_{ij}/N_i$. Then $d_{ij} \dot{\sim} N(\frac{p_j * c_{ij}}{\Sigma_{j'} p_{j'} * c_{ij'}}, \frac{p_j * c_{ij}}{N_i \Sigma_{j'} p_{j'} * c_{ij'}})$.

Our model assumes each cell is associated with one of K clusters. Each cell is also associated with a latent variable $s_i \in [1,2]$ (or *s-score*) defining its time within S-phase. Given hyper parameters *L* and *R* we define *L* replication regime functions:

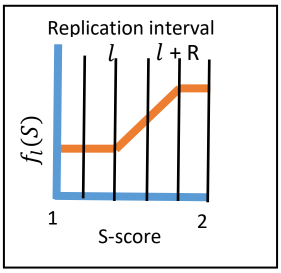

$f_l(s) = 2 + 2 * \min(\max((s - \mathrm{repl\_start}_l)/(\mathrm{repl\_end}_l - \mathrm{repl\_start}_l), 0), 1)$, where:

$\mathrm{repl\_start}_l = 1 + \frac{l-1}{L+R-1}$ and $\mathrm{repl\_end}_l = 1 + \frac{l+R-1}{L+R-1}$

as illustrated in the right figure. The vector $r_j(k)$ designates the replication regime $\in \{1, \dots, L\}$ of bin j in cluster k. The copy numbers of bin j for cell i from cluster k is given by $c_{ij} = f_{r_j(k)}(s_i)$.

These copy numbers now define the probabilistic model per cell cluster:

$$\Pr\left(d_{ij} \mid s_i, r_j(k), p_j\right) \dot{\sim} N\left(\frac{p_j * f_{r_j(k)}(s_i)}{\Sigma_{j'} p_{j'} * f_{r_{j'}(k)}(s_i)}, \frac{p_j * f_{r_j(k)}(s_i)}{N_i \Sigma_{j'} p_{j'} * f_{r_{j'}(k)}(s_i)}\right).$$

Finally, the mixture model is defined by placing probabilities ϕ_k as the priors on association of each cell with cluster k. The fully parametric generative model for D can now be formulated using s-scores (s), cluster association (k), cluster replication regime association $r_j(k)$, cluster priors (ϕ_k) and bins coverage prior probabilities p_j:

$$\log(\Pr\left(D|s, k, r_j, \phi_k, p_j\right)) = \sum_{ij} \log\phi_{k(i)} + \log\Pr(d_{ij}|s_i, r_j\left(k(i)\right), p_j)$$

***3. Mixture model inference and parameter optimization*.**

We use an EM-like algorithm to fit our mixture model. Denote by z_{ik} the latent indicator variable that cell i belongs to cluster k. Denote by D the observed data, by Θ all the parameters of the model and by Θ^t their current estimates. We also add a *characteristic replication time* parameter r_j that is fitted and used for regularization as described below.

Initialization: initial values of $E[z_{ik}]$ and s_i are expected as input.

E-step: The E step of the algorithm computes z_{ik}'s expected value:

$$\begin{aligned} E[z_{ik}] &= \Pr(cell\ i\ belongs\ to\ cluster\ k \mid D, \Theta^t) \\ &\propto \phi_k \Pr(D|cell\ i\ belongs\ to\ cluster\ k, \Theta^t) \\ &= \phi_k \Pi_j N\left(d_{ij}, \frac{p_j * f_{r_j(k)}(s_i)}{\Sigma_{j'} p_{j'} * f_{r_{j'}(k)}(s_i)}, \frac{p_j * f_{r_j(k)}(s_i)}{\Sigma_{j'} p_{j'} * f_{r_{j'}(k)}(s_i)}\right) = z'_{ik} \end{aligned}$$

For increased numerical stability, we compute $\log(z'_{ik})$, and calculate $E[z_{ik}] = \frac{1}{\Sigma_{k'} \exp(\log(z'_{ik'}) - \log(z'_{ik}))} = \frac{z'_{ik}}{\Sigma_{k'} z'_{ik'}}$

M-step: in this step we optimize the expected complete likelihood function with regularization:

$$Q(\Theta|\Theta^t) = \Sigma_{i,j,k}E[z_{ik}] * \left[\log(\phi_k) + \log\left(N\left(d_{ij}, \frac{p_j * f_{r_j(k)}(s_i)}{\Sigma_{j'}p_{j'} * f_{r_{j'}(k)}(s_i)}, \frac{p_j * f_{r_j(k)}(s_i)}{N_i\Sigma_{j'}p_{j'} * f_{r_{j'}(k)}(s_i)}\right)\right)\right] + \lambda\Sigma_{j,k}|r_j(k) - r_j|$$

r_j is a parameter signifying a characteristic replication time regime for bin j, and the regularization term $\lambda\Sigma_k|r_j(k) - r_j|$ is used to penalize drift of cluster-specific replication regimes. λ is a parameter that controls the strength of the penalty. This regularization term ensures that the s-scores in different clusters are generally synchronized.

To optimize Q we proceed by alternating between optimization of some parameters while fixing all others, using the following step types:

M1: Optimize ϕ_k. The optimization problem for ϕ_k is standard for mixture models, and results in the update rule: $\phi_k = \Sigma_i E[z_{ik}]/n$, where n is the number of cells.

M2: Optimize s_i. The optimization problem for s_i is independent between cells, so each s_i can be optimized separately by grid search (covering the range [1:2])

M3: Optimize r_j. The optimization of the r_j values is also independent between genomic bins. We therefore optimize each bin separately by enumerating over all discrete *L* possibilities.

M4: Optimize $r_j(k)$. Here bins are not independent since they appear in the denominator in the expression: $\frac{p_j * f_{r_j(k)}(s_i)}{\Sigma_{j'}p_{j'} * f_{r_{j'}(k)}(s_i)}$. However, the effect of each bin on the denominator is small, due to the large number of bins. We therefore first optimize all $r_j(k)$ while assuming fixed denominator, update the denominator, and repeat this process until the fraction of $r_j(k)$ values that change is smaller than 1/1000. During the first iteration of the algorithm, since initial $r_j(k)$ values are not given as input, we assume that the denominator increases linearly from 2 to 4 as a function of s_i.

M5: We re-optimize $r_j(k)$ sequentially without assuming a constant denominator until convergence (which is defined by having less than 1/1000 of the bins changed).

Generalized EM. We combine the steps described above by iterating on the following sequence of optimization steps:

Iterate{M3, M4, M5, E, M1, M2, E}

At the end of each iteration we cluster the cells as follows: $\text{cluster}(\text{cell } i) = \text{argmax}_k E[z_{ik}]$.

The algorithm stops when the fraction of cells that changed clusters is ≤ 0.01, and the fraction of $r_j(k)$ values that changed is ≤ 0.001.

4. Simulations and cross-validations for the mixture model.

Simulations: We simulated 240 cells with 9600 bins, originating from 5 clusters. The bins had 3 different replication regimes and the replication rate was 1 ($L = 3, R = 1$). 50% of the bins had the same replication timings in all clusters, with an equal number of them in each of the replication timings. 25% of the bins started replication at the second interval in all clusters except for one cluster, where they replicated at the first interval. These 25% of bins were equally divided between the 5 clusters. Similarly, 25% of the bins replicated in the third interval except for one cluster where they replicated in the second interval.

In the simulation we sampled reads from each cell such that on average each genomic bin had 10 reads. We sampled the reads from a multinomial distribution, such that the probability of each bin to be sampled is proportional to its actual DNA content as defined by the model. That is, a read from bin j was sampled with probability $\frac{f_{r_j(k)}(s_i)}{\sum_{j'} f_{r_{j'}(k)}(s_i)}$.

*Cross validation***.** To validate the model, and in order to select hyperparameters (R, L, λ and the number of clusters), we performed cross validation on the data. In the outer iteration we hold out one chromosome at a time and learn a model using all other chromosomes. For each such model, the cells are partitioned randomly into 10 folds. For each fold, we perform a single M step to learn $r_j(k)$ for all bins j in the left-out chromosome, without using the cells that belong to the held-out fold. For every left-out chromosome and cell fold, the model now has estimates for s_i of the fold's cells, and for $r_j(k)$ for all bins in the held-out chromosome. We use these parameters and the generative model to predict the data in the held-out chromosome for the held-out cells and compare the prediction to the observed (but held-out) data. To assess the performance of the model, after the model predicts values for all cell folds, we calculate the correlation between the observed and predicted values. This process is repeated for all chromosomes. The final performance of the model is the mean of all these correlations across all chromosomes.

5. Extending mixture model clusters to more cells.

We applied the mixture model clustering to a subset of the cells which are in mid-S phase. We describe a procedure for robust expansion of the derived clusters to additional cells, improving the sensitivity of the S-phase seeding algorithm that we described above.

i) We define genomic bin clusters $J_k = \{j \; s.t. \; r_j(k) - \mathrm{mean}_{k'}(r_j(k')) < -T\}$.

ii) We compute a trans A-score for each cell and bin cluster, analogously to the $\mathrm{cell\_A}$ values defined above, but using trans-chromosomal contacts instead of long-range cis contacts:

$$\mathrm{cell\_trA}_k^i = \sum_{\{j \in J_k\}} trA_j^i / \left[\sum_{\{j \in J_k\}} trA_j^i + \sum_{\{j \in J_k\}} trB_j^i\right]$$

Using only inter-chromosomal contact helps to control for the effect of the change in contact distance distribution during S-phase.

iii) To control for the effect of increase in A compartment strength during the cell cycle, we normalize the observed trans A-score using shuffling controls. This is done by subtracting from the score the mean of $\mathrm{cell\_trA}_k^i$ in randomized J_k bin clusters. We make sure randomized bin clusters match the A-score distribution of the group J_k.

iv) We represent every cell by the normalized vector $\mathrm{cell\_trA}_k^i$ across all clusters. For every cluster k, we then train a linear SVM model to predict whether a cell belongs to cluster k or not. The models are trained with the cells used as input for the mixture model. We apply all cluster classifiers to all cells, and classify only cells that were classified to exactly one cluster.

6. Using the mixture model to normalize the effect of the cell cycle on coverage

Given the inferred s-scores, we calculated d_{ij}/p_j as the G1-normalized coverage matrix. We then ordered cells by their inferred s-score, and subtracted from the G1-normalized coverage of each bin its running mean using 20 cells.
