# Supplementary material for: Single cell Hi-C identifies plastic chromosome conformations underlying the gastrulation enhancer landscape
Source: Nat Commun. 2023 Jun 29;14:3844. doi: 10.1038/s41467-023-39549-4 (PMC10310791; doi:10.1038/s41467-023-39549-4)
Supplement: Supplementary file 3 — Description of Additional Supplementary Files [file 41467_2023_39549_MOESM3_ESM.pdf]

Description of Additional Supplementary Files

File Name: Supplementary Data 1
Description: Differences in chromosomal conformation of ESC and E9 embryo cells, for genes that are differentially expressed between the ESC and the embryo. Rows represent different genes, and the difference in the shaman score of the gene's transcription start site between ESC and embryo is listed for different genomic distances.

File Name: Supplementary Data 2
Description: Genomic bins with high A-score differences between mesoderm and ectoderm. The bin's coordinates, A-score in mesoderm and ectoderm, and contained genes, are listed in each row.

File Name: Supplementary Data 3
Description: Promoter-enhancer pairs with three-way support for ectoderm or mesoderm regulatory activity. Each row represents a pair, and lists the gene's coordinates, the enhancer's coordinates, and the shaman score for the pair in mesoderm and ectoderm.

File Name: Supplementary Data 4
Description: Promoter- H3K27me3 locus pairs with three-way support for ectoderm or mesoderm regulatory activity. Each row represents a pair, and lists the gene's coordinates, the H3K27me3 locus' coordinates, and the shaman score for the pair in mesoderm and ectoderm.
