# Supplementary material for: Single cell Hi-C identifies plastic chromosome conformations underlying the gastrulation enhancer landscape
Source: Nat Commun. 2023 Jun 29;14:3844. doi: 10.1038/s41467-023-39549-4 (PMC10310791; doi:10.1038/s41467-023-39549-4)
Supplement: Supplementary file 8 — Reporting Summary [file 41467_2023_39549_MOESM8_ESM.pdf]

nature portfolio

Corresponding author(s): Amos Tanay

Last updated by author(s): May 20, 2023

Reporting Summary

Nature Portfolio wishes to improve the reproducibility of the work that we publish. This form provides structure for consistency and transparency in reporting. For further information on Nature Portfolio policies, see our Editorial Policies and the Editorial Policy Checklist.

Statistics

For all statistical analyses, confirm that the following items are present in the figure legend, table legend, main text, or Methods section.

n/a	Confirmed	
☐	☒	The exact sample size (*n*) for each experimental group/condition, given as a discrete number and unit of measurement
☐	☒	A statement on whether measurements were taken from distinct samples or whether the same sample was measured repeatedly
☐	☒	The statistical test(s) used AND whether they are one- or two-sided<br>*Only common tests should be described solely by name; describe more complex techniques in the Methods section.*
☐	☒	A description of all covariates tested
☐	☒	A description of any assumptions or corrections, such as tests of normality and adjustment for multiple comparisons
☐	☒	A full description of the statistical parameters including central tendency (e.g. means) or other basic estimates (e.g. regression coefficient) AND variation (e.g. standard deviation) or associated estimates of uncertainty (e.g. confidence intervals)
☐	☒	For null hypothesis testing, the test statistic (e.g. *F*, *t*, *r*) with confidence intervals, effect sizes, degrees of freedom and *P* value noted<br>*Give P values as exact values whenever suitable.*
☒	☐	For Bayesian analysis, information on the choice of priors and Markov chain Monte Carlo settings
☐	☒	For hierarchical and complex designs, identification of the appropriate level for tests and full reporting of outcomes
☐	☒	Estimates of effect sizes (e.g. Cohen's *d*, Pearson's *r*), indicating how they were calculated

Our web collection on statistics for biologists contains articles on many of the points above.

Software and code

Policy information about availability of computer code

Data collection	No software was used for data collection.
Data analysis	We used custom code for the analysis, which is available in: https://github.com/tanaylab/scHiC_embryo<br>Our analysis included use of the following versions of software packages:<br>shaman - 2.0<br>misha - 4.0.10<br>metacell (Baran et al.) - 0.3.41<br>metacell 2 (Ben-Kiki et al.) - 0.8.0

For manuscripts utilizing custom algorithms or software that are central to the research but not yet described in published literature, software must be made available to editors and reviewers. We strongly encourage code deposition in a community repository (e.g. GitHub). See the Nature Portfolio guidelines for submitting code & software for further information.

Data

Policy information about availability of data

All manuscripts must include a data availability statement. This statement should provide the following information, where applicable:

- Accession codes, unique identifiers, or web links for publicly available datasets
- A description of any restrictions on data availability
- For clinical datasets or third party data, please ensure that the statement adheres to our policy

The scHi-C and scRNA-seq data generated in this study have been deposited in the GEO database under accession code GSE148793. The ESC scHi-C data used in this study are available in the GEO database under accession code GSE94489. The previously published embryo gastrulation scRNA-seq data used in this study are available in the ArrayExpress database under accession code E-MTAB-6967. The scRNA / scATAC multiome data used in this study are available in the GEO database under accession code GSE205117. The neural progenitor cells' Hi-C data used in this study are available in the GEO database under accession code GSE96107. The hematopoietic Hi-C data used in this study are available in the GEO database under accession code GSE119201.

Research involving human participants, their data, or biological material

Policy information about studies with human participants or human data. See also policy information about sex, gender (identity/presentation), and sexual orientation and race, ethnicity and racism.

Reporting on sex and gender	We didn't collect or analyze any human data in this study.
Reporting on race, ethnicity, or other socially relevant groupings	We didn't collect or analyze any human data in this study.
Population characteristics	We didn't collect or analyze any human data in this study.
Recruitment	We didn't collect or analyze any human data in this study.
Ethics oversight	We didn't collect or analyze any human data in this study.

Note that full information on the approval of the study protocol must also be provided in the manuscript.

Field-specific reporting

Please select the one below that is the best fit for your research. If you are not sure, read the appropriate sections before making your selection.

☒ Life sciences ☐ Behavioural & social sciences ☐ Ecological, evolutionary & environmental sciences

For a reference copy of the document with all sections, see nature.com/documents/nr-reporting-summary-flat.pdf

Life sciences study design

All studies must disclose on these points even when the disclosure is negative.

Sample size	Cells from embryos of three mice were used for generating the single-cell Hi-C library. Cells from embryos of two pregnant mice were used for generating single-cell RNA-seq libraries. We did not perform calculations to select the sample size, but due to the difficulty and high resource requirements of the performed experiments we were limited to a small number of embryos. We therefore used a small number, that is still higher than one, so that the results will represent more than a single embryo.
Data exclusions	Cells that did not pass quality control (QC) were excluded from the analysis. The criteria for QC for the Hi-C data include the number of reads per cell and the maximal chromosomal aberration. Full criteria are listed in the Methods section. The criteria for QC for the scRNA-seq data include a minimal number of UMIs.
Replication	To validate the distinct chromosomal conformation of the primitive Erythrocytes, we sorted for these cells and observed the same distinct chromosomal conformation (as shown in Supplementary Figure 5). We performed one such validation experiment. We did not perform replication experiments for other results presented in this study.
Randomization	No randomization was performed in this study. Randomization was not relevant for this study, as our study describes the genome organization of the genome in mouse embryos and does not contain a treatment or any group allocation.
Blinding	No blinding was performed in this study. Blinding was not relevant for this study, as our study describes the genome organization of the genome in mouse embryos and does not contain a treatment or any group allocation.

Reporting for specific materials, systems and methods

We require information from authors about some types of materials, experimental systems and methods used in many studies. Here, indicate whether each material, system or method listed is relevant to your study. If you are not sure if a list item applies to your research, read the appropriate section before selecting a response.

Materials & experimental systems

n/a	Involved in the study
☐	☒ Antibodies
☒	☐ Eukaryotic cell lines
☒	☐ Palaeontology and archaeology
☐	☒ Animals and other organisms
☒	☐ Clinical data
☒	☐ Dual use research of concern
☒	☐ Plants

Methods

n/a	Involved in the study
☒	☐ ChIP-seq
☐	☒ Flow cytometry
☒	☐ MRI-based neuroimaging

Antibodies

Antibodies used	anti-CD71-PE (Invitrogen 12-0711-82), anti-TER119-APC (Invitrogen 17-5921-82)
Validation	Both antibodies were previously used in: https://doi.org/10.1182/blood-2006-03-006569

Animals and other research organisms

Policy information about studies involving animals; ARRIVE guidelines recommended for reporting animal research, and Sex and Gender in Research

Laboratory animals	Two 129S4/SvJae pregnant female mice were sacrificed for the single-cell RNA-seq dataset. Three C57BL/6 wildtype pregnant female mice were sacrificed for the single-cell Hi-C dataset. One C57BL/6 pregnant, female mice was sacrificed for the primitive erythrocytes single-cell Hi-C dataset.
Wild animals	The study did not involve wild animals.
Reporting on sex	The sex of the embryos was not collected, and the cells from multiple embryos were combined together. The small number of samples does not allow a statistically robust investigation into sex differences.
Field-collected samples	The study did not involve samples collected from the field.
Ethics oversight	Animal committees at the Babraham Institute, the Florida State University Animal Care and Use Committee (ACUC), and the Weizmann Institute of Science approved and provided guidance for this study.

Note that full information on the approval of the study protocol must also be provided in the manuscript.

Flow Cytometry

Plots

Confirm that:

☒ The axis labels state the marker and fluorochrome used (e.g. CD4-FITC).

☒ The axis scales are clearly visible. Include numbers along axes only for bottom left plot of group (a 'group' is an analysis of identical markers).

☒ All plots are contour plots with outliers or pseudocolor plots.

☒ A numerical value for number of cells or percentage (with statistics) is provided.

Methodology

Sample preparation	Cells were thawed and stained for CD71and TER119. Cells were first blocked with 1mL of PBS-FT (5% FBS, 0.1% Tween-20) for 1 hour, then stained with 1:200 anti-CD71-PE (Invitrogen, 12-0711-82) and 1:200 anti-TER119-APC (Invitrogen, 17-5921-82) for 2 hours at room temperature. Cells were washed and resuspended in 500uL PBS-F (2% FBS) and Hoechst (15ug/mL) and subjected to FACS by Aria (BD Biosciences). Primitive erythrocytes (CD71+, TER119+) were collected and pooled into a 50mL falcon for scHi-C processing.
Instrument	Aria (BD Biosciences)
Software	We used no custom code to analyzing the flow cytometry data.

Cell population abundance	CD71+/Ter119+ (P2) cells were 81% of the single cell population (P1). Single cells (P1), identified via Hoechst staining, was 94.3% of the overall population.
Gating strategy	Debris was gated out based on Hoechst staining. CD71+/TER119+ cells were then gated for collection.

☒ Tick this box to confirm that a figure exemplifying the gating strategy is provided in the Supplementary Information.
